# A Monte Carlo Evaluation of Weighted Community Detection Algorithms

**DOI:** 10.3389/fninf.2016.00045

**Published:** 2016-11-10

**Authors:** Kathleen M. Gates, Teague Henry, Doug Steinley, Damien A. Fair

**Affiliations:** ^1^Department of Psychology, University of North CarolinaChapel Hill, NC, USA; ^2^Department of Psychological Sciences, University of MissouriColumbia, MO, USA; ^3^Departments of Behavioral Neuroscience and Psychiatry, Oregon Health and Sciences UniversityPortland, OR, USA

**Keywords:** community detection, modules, functional connectivity, functional MRI, Monte Carlo simulation

## Abstract

The past decade has been marked with a proliferation of community detection algorithms that aim to organize nodes (e.g., individuals, brain regions, variables) into modular structures that indicate subgroups, clusters, or communities. Motivated by the emergence of big data across many fields of inquiry, these methodological developments have primarily focused on the detection of communities of nodes from matrices that are very large. However, it remains unknown if the algorithms can reliably detect communities in smaller graph sizes (i.e., 1000 nodes and fewer) which are commonly used in brain research. More importantly, these algorithms have predominantly been tested only on binary or sparse count matrices and it remains unclear the degree to which the algorithms can recover community structure for different types of matrices, such as the often used cross-correlation matrices representing functional connectivity across predefined brain regions. Of the publicly available approaches for weighted graphs that can detect communities in graph sizes of at least 1000, prior research has demonstrated that Newman's spectral approach (i.e., Leading Eigenvalue), Walktrap, Fast Modularity, the Louvain method (i.e., multilevel community method), Label Propagation, and Infomap all recover communities exceptionally well in certain circumstances. The purpose of the present Monte Carlo simulation study is to test these methods across a large number of conditions, including varied graph sizes and types of matrix (sparse count, correlation, and reflected Euclidean distance), to identify which algorithm is optimal for specific types of data matrices. The results indicate that when the data are in the form of sparse count networks (such as those seen in diffusion tensor imaging), Label Propagation and Walktrap surfaced as the most reliable methods for community detection. For dense, weighted networks such as correlation matrices capturing functional connectivity, Walktrap consistently outperformed the other approaches for recovering communities.

## 1. Introduction

Network theory has been used to examine the organization of networks across disparate research foci, including the World Wide Web (Latora and Marchiori, 2001), social networks (Zachary, 1977), the power grid (Watts and Strogatz, 1998), brain processes (Bassett and Bullmore, 2006), and food-webs (Dunne et al., 2002). It has been shown in research using both structural and functional brain data that networks of the brain exist that have well-defined internal structures that can be described or demarcated with network theory analyses (Sporns, 2011). For this reason, neuroscientists often organize brain regions into neural networks from within a network theoretic perspective (e.g., McNally et al., 2011) using community detection. While work on methods for arriving at community structures in networks began in 1927 (Rice, 1927), there has been a proliferation of techniques in the short time since Girvan and Newman's seminal paper (Girvan and Newman, 2002). This paper introduced a highly successful function (“modularity”) for arriving at a stopping point within the community structure search (Fortunato, 2010). An unintended consequence of this influx is that investigators use algorithms that work well on one type of data or problem (e.g., count matrices; large graphs) on other types of data (e.g., correlation matrices; smaller graphs) for which they have not been evaluated (Orman and Labatut, 2009). This is particularly evident in neuroimaging studies where community detection is used on functional connectivity correlation matrices obtained from fMRI data (e.g., Mumford et al., 2010; Rubinov and Sporns, 2010) as well as on matrices generated from diffusion tensor imaging (DTI; e.g., Bassett et al., 2011). In both cases, the goal is to uncover neural components that are highly connected to others in their module and less connected to components outside their module. However, at present it is unknown the extent to which a given weighted community detection algorithms perform well when applied to correlation matrices that neural scientists often use (Orman and Labatut, 2009). The distributional differences in data types (i.e., count vs. correlational matrices) are likely to have an effect on the performance of a given algorithm. To assist in selecting the appropriate algorithm given the qualities of the data, the present paper offers formal and independent testing of the most commonly used algorithms across different data formats and numerous plausible conditions that likely will be encounter by neural scientists.

For generalizability when describing the community detection algorithms we offer some terms. Graphs (“networks”) can generally be defined as a set of nodes (also called “vertices”) that are connected in pairs by edges (“adjacencies,” “connections,” or “ties”). “Degree” generally refers to the count of the number of connections a node has; for correlation and Euclidean distances, this is technically called “strength.” Connections among nodes within the same community are quantified with “in-degree,” with connections for nodes in different communities is termed “out-degree.” To enable succinctness in writing, we use in-degree and out-degree even when discussing weighted networks since the concepts of degree and strength are synonymous. Practically, graphs are simply square matrices that in some way indicate the degrees of connectivity among the brain regions or voxels. Most commonly, in neuroimaging studies each element in the matrix (i.e., edge) indicates the connectivity weight (either functional or structural). Communities are formally considered to be subgroups of nodes comprising a modular component that, in terms of edges, has higher within-community connectivity than expected by chance given the properties of the network as a whole (Guimerà et al., 2005; Newman, 2006). One particularly useful benefit of many community detection techniques is that the researcher does not need to specify *a priori* the number of communities (or “clusters,” “modules,” “classes,” or “subgroups”).

Community detection approaches arose from the inability to feasibly and efficiently compare all possible partitions for a given set of data, including relatively small graphs. The presence of large-scale data collection, greater computing power, and a desire to understand behaviors of systems as a whole has altered the way in which networks are approached (Albert and Barabási, 2002). Since the number of possible partitions grows faster than the exponential rate, the most recently developed algorithms aim to partition even larger sets of nodes with greater computational efficiency. While these methods have demonstrated success in their ability to recover true community structures with relatively low error in large networks, they have not all been evaluated on smaller systems (e.g., Infomap; Rosvall and Bergstrom, 2008). Given the exceptional performance on large graphs (on the order of thousands), neuroimagers routinely apply community detection algorithms to graphs with node size on the order of hundreds (e.g., Power et al., 2011) and smaller (e.g., He et al., 2009). This disconnect between evaluation on large graphs and use on smaller graphs needs to be rectified so researchers have adequate information with which to identify algorithms that perform well at this smaller resolution. A primary aim of the current paper is to test the use of these methods on smaller network sizes to understand the robustness across varying data types.

Of perhaps greater importance is that there is a gap in the literature such that community detection algorithms have typically been developed and tested on binary matrices or the weighted counterpart: sparse count matrices. In practice, however, these algorithms are increasingly being used on networks quantified as correlation matrices for which they have not been evaluated. This regularly occurs in studies analyzing fMRI data, where the temporal bivariate correlations across time among predefined brain regions constitute the graph (e.g., Fair et al., 2009). What is often overlooked is that correlation matrices have distinct properties that are unlike those seen in sparse count matrices. In addition to being dense, there are explicit node interdependencies. These are not taken into account in any of the weighted algorithms, most of which assume that the weight of an edge between two nodes A and B is independent of the weight of edges between node A and other nodes (i.e., dyadic independence exists). Still, some algorithms might perform well on correlation matrices despite being developed for use with sparse matrices—others may not. The present paper explores which community detection algorithms perform most reliably on graphs conceived as similarity matrices, such as correlation and the reflected difference measure Euclidean distance, which could feasibly be generated for networks such as brain regions of interest.

The major contribution provided by the present paper is the independent evaluation of community detection algorithms' ability to assign nodes to their true community for sparse count, correlation, and reflected Euclidean distance (EucD) matrices across a number of conditions using simulated data. The most popular algorithms have predominantly been evaluated using empirical data (Clauset et al., 2004; Newman, 2006; Blondel et al., 2008). To date, few large-scale independent study have compared approaches across numerous varied conditions using simulated data (Schaub et al., 2012), and none have focused on the use of correlation matrices (Pons and Latapy, 2006; Orman and Labatut, 2009; Lancichinetti and Fortunato, 2011, all generated count matrices). Importantly, our work tests these varied matrix types across parameters and conditions that are seen in empirical brain data across varied samples and likely to influence the efficacy of community detection algorithms. Explained in detail below, the simulations broadly vary across these conditions: (1) graph size; (2) heterogeneity in node edge weights within communities; (3) community sizes; (4) number of communities; (5) highly correlated communities (i.e., ill-defined communities); and (6) between-community heterogeneity in edge weight (i.e., level differences according to community). Following an overview of the methods tested, we provide a description of the creation of these benchmark graphs. Next, we present results for each method applied to each graph. We conclude with summaries of the conditions in which each one thrived (and failed) at recovering the true community structures and suggestions for users. The present paper provides an unbiased, direct comparisons of publicly distributed community detection approaches for undirected, weighted graphs under varied conditions often found in real networks.

## 2. Methods

### 2.1. Community detection algorithms tested

We do not explore unweighted, directed, or graph-partition approaches. While many unweighted approaches can be extended to accommodate weighted matrix entries, we only evaluated approaches that already have a weighted algorithm that is publicly distributed. This directly follows from the primary goal of the present paper, which is to evaluate commonly used algorithms under various conditions that previously have not been explored. Directed approaches (i.e., asymmetric matrices) pertain to specific topics that as of yet, have not reached the popularity of undirected graphs in neuroimaging studies, and thus we do not examine them in detail. Also, unlike the alterations needed to make an unweighted approach accommodate weights, it is less straightforward to make undirected approaches work for directed graphs (Fortunato, 2010). However, as the popularity of community partitions in direct graphs increases a thorough examination, as done here, will be warranted. The class of algorithms under graph-partition approaches require knowledge of the number of groups, which is usually not known. We test only methods for which *a priori* information regarding the community structure (including number of partitions) is not necessary. We also limited our investigation to algorithms that can accommodate >75 nodes and do not allow for overlap in communities (such as OSLOM; Lancichinetti et al., 2011). This left 6 popular approaches: Newman's Spectral Approach (also referred to as Leading Eigenvector), Walktrap, Fast Modularity, the Louvain method (i.e., the multilevel community method), Label Propagation, and Infomap. These approaches have the following properties: (1) have been extended for use on weighted graphs; (2) can perform on graphs with node size >75; (3) assume the graph is undirected (i.e., symmetric); (4) do not require the user to indicate the number of communities. The specific sources for these algorithms are listed in Table 1.

**Table 1:** **Community detection methods tested**.

**Method**	**Platform**	**Function**	**Source**
NSA	Matlab Toolbox	modularity_und.m	https://sites.google.com/site/bctnet/
Walktrap	R Package	walktrap.community	igraph; CRAN
Fast Modularity	R Package	fastgreedy.community	igraph; CRAN
Louvain Method	Matlab Toolbox	modularity_louvain_und_sign.m	https://sites.google.com/site/bctnet/
Label Propagation	R Package	label.propagation.community	igraph; CRAN
Infomap	R Package	infomap program	http://www.mapequation.org/

We briefly discuss the algorithms selected for comparison. Technical descriptions have been extensively discussed in reviews (Porter et al., 2009; Fortunato, 2010) as well as specific sources cited below and thus are not repeated in detail here. The present study seeks to evaluate algorithms and thus does not adapt or change them from the original authors. For instance, some algorithms include negative values whereas others set these to zero. We use the algorithms as developed by the originators and do not alter these decisions so that results directly speak to the present algorithms' ability to detect communities across conditions. Another difference is that some algorithms are deterministic, meaning that each run on the same data will produce the same community assignments, whereas others (such as the Louvain GJA method and Label Propagation), are not deterministic. While the authors of these algorithms suggest conducting multiple runs to identify similarities in community assignments, the main findings here present the results when running the algorithm once on each data set. The rationale being that we wish to test the algorithm in its current form and not have our results influenced by an algorithm we would introduce to consolidate the findings across multiple runs. In an auxiliary set of results we provide results for when we ran Louvain GJA and Label Propagation 100 times on each data set. For these results one can identify the degree to which the solutions differ across runs and if this relates to accuracy in findings.

One striking commonality is that Newman's Spectral algorithm, Fast Modularity, and the Louvain methods are hierarchical clustering approaches that maximize a score called, modularity, and Walktrap uses this score to identify the optimal partitioning of nodes. Despite this similarity, the reader will soon see that the results vary greatly.

In weighted networks, modularity measures the density (or strength, when the matrices are not count) of edges within a community compared against the density (strength) of edges outside the community (Blondel et al., 2008). When the strength of edges within a group is larger than what is expected at random, modularity rises (Newman and Girvan, 2003; Newman, 2004a). Formally, modularity for weighted networks can be written as (Fan et al., 2007):

(1) $$\begin{array}{l} Q^{w}=\frac{1}{2w}\underset{ij}{\sum }\left(w_{ij}-\frac{w_{i}w_{j}}{2w}\right)\delta \left(s_{i},s_{j}\right) \end{array}$$

with δ being 1 if the nodes belong to the same community and 0 if otherwise. The value *w*_*i*_*w*_*j*_/*2w* provides the expected strength of the edge between the given nodes with *w* being the summation of the edge strengths in the network and *w*_*i*_ (*w*_*j*_) the summed strength of node *i* (*j*). *w*_*ij*_ is the edge strength for the provided node pair. Maximizing modularity can be used to arrive at the best partitioning of communities with small and negative numbers indicating no clear communities. For methods that maximize modularity, the division (or combining of communities in agglomerative approaches) of communities stops when there no longer is a positive change in modularity. Hence the definition of the community for modularity is that they are indivisible subgraphs (Newman, 2006). Modularity has surpassed other metrics in its ability to identify the optimal partition of communities (Danon et al., 2006); however, it comes with limitations such as a reported inability to recover graphs with small communities (Fortunato and Barthélemy, 2007; Porter et al., 2009), different sized communities (Pons and Latapy, 2006), and sometimes inflated modularity for sub-optimal partitions (Fortunato, 2010).

#### 2.1.1. Newman's spectral approach (NSA)

Newman (2006) introduced a spectral approach that iteratively places nodes into communities based on the principal eigenvector after decomposition of a modularity matrix, which has some similar properties to the commonly used Laplacian graph. In short, the modularity matrix results from subtracting the degree (equivalently the “weight”) that is expected by chance between two given nodes from their actual edge weight. Nodes that have positive loadings on this first component are placed in one group, with nodes having negative loadings placed in another. From there groups are iteratively created in this divisive manner until there is no longer any improvement in modularity. When compared to prior approaches that maximized modularity, this approach obtained the highest modularity on empirical data sets that were count matrices (Newman, 2006). This was taken to mean that the quality of the partitions was better. It should be noted that modularity has previously been suggested to not be a poor marker of performance (Porter et al., 2009). Simulated data comparisons conducted on sparse count matrices suggest that NSA may not perform optimally when the ratio of internal to external community degree is low (Orman and Labatut, 2009).

#### 2.1.2. Walktrap

A random walk approach, Walktrap (Pons and Latapy, 2006), which is related to Newman (2006)'s eigendecomposition algorithm described above, enables greater computational efficiency without sacrificing reliability of results. Walktrap begins by generating a matrix of transition probabilities for each pair of nodes. Each element of this matrix represents the transition probability of going from one node to each given node in a random walk of a given length of time. The transition probabilities, which are based on node degrees or strength (i.e., average weight of edges from the given node to other nodes), are used to arrive at a distance measure for each pair of nodes. A traditional hierarchical clustering technique (Ward's method; Ward, 1963) is then applied to this distance matrix, forming communities by minimizing the sum of squared distances of each node to the other nodes within its own community. Conceptually, this approach aims to arrive at communities that have more transitions within clusters than with outside nodes. This agglomerative approach utilizes modularity to select the optimal partition. Prior work on simulated sparse count matrices has suggested that Walktrap performs well in the presence of lower in-degree to out-degree ratios (Orman and Labatut, 2009) and at graph sizes as small as 100 (Pons and Latapy, 2006), particularly as compared to methods which maximize modularity.

#### 2.1.3. Fast modularity

**Figure 1:**
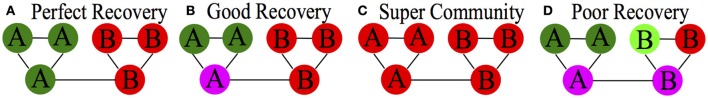
**Conceptual depiction of types of accuracy**. **(A)** “Perfect Recovery” refers to all nodes being placed in the correct community; **(B)** “Good Recovery” reflects nodes being placed in the correct community most of the time; **(C)** “Super Community” indicates a phenomenon where nodes are subsumed into one large community; **(D)** “Poor Recovery” is when nodes are simply placed in communities with other nodes erroneously. **(A)** indicates ARIHA of 1.00; **(B)** would have < 1.00; **(C,D)** would be closer to 0.00.

Fast modularity scales up the ability of a previous greedy algorithm (Newman, 2004b) for use on matrices as large as 10 million (Clauset et al., 2004). The agglomerative algorithm merges together communities, starting with each node as its own community, in order to optimize modularity. Fast modularity capitalizes on shortcuts based on the premise that sparse graphs are being used. However, in the absence of sparsity the algorithm still should perform well (albeit slower). A drawback of fast modularity comes from its propensity to produce super communities that contain a large fraction of nodes (Fortunato and Barthélemy, 2007) as depicted in Figure 1 and may not perform well for graph as small as 100 (Pons and Latapy, 2006).

#### 2.1.4. Louvain GJA method

What has come to be known as the Louvain method (or the “multilevel community method” developed by Blondel and colleagues) similarly utilizes modularity to identify the best partitions but uses a different method than the previously described approaches (Blondel et al., 2008; Rubinov and Sporns, 2011). Importantly, the motivation behind the Louvain method was to reveal the hierarchical structure of very large graphs (hundreds of millions). It identifies the local maxima of modularity by looking iteratively at the change in modularity that results from moving each node to another community, with each node starting in its own community. The algorithm iteratively creates smaller weighted networks by creating latent “supernodes” generated by the nodes within a community and identifying edge weights between these latent nodes and other observed or latent nodes. In this way, the Louvain method claims to circumvent the aforementioned resolution problem seen in modularity-optimization approach in that it can find small communities. We present here results from the GJA algorithm (Rubinov and Sporns, 2011) which offers improvements upon the original algorithm as described by Blondel et al. (2008).

#### 2.1.5. Label propagation

Label propagation (Raghavan et al., 2007) iteratively assigns community designations by placing nodes in a community that contains the majority of its neighbors. An important note is that the algorithm begins by randomly placing individuals into communities; thus multiple runs on the same data may remit different solutions and the ordering may have implications for the reliability of the solution (Steinley, 2003). The aim is to arrive at communities in the strong sense, with each node having more edges with others in its community than those out of their community. Because of this constraint, label propagation may work better on sparse matrices. This is a fast technique that works well on large matrices. It is equivalent to finding the local energy minima of a simple zero-temperature Potts model, but the number of minima is larger than the number of nodes (Tibély and Kertész, 2008). Label Propagation was shown in simulated sparse count matrices to evidence a steep decline in performance once the weight of in-degree to out-degree became more equivalent (Orman and Labatut, 2009).

#### 2.1.6. Infomap

Rosvall and Bergstrom (2008) developed an approach that, like Walktrap, utilizes random walks. This algorithm also uses the definition that there should be minimal transitions of a random walker between clusters and more transitions within clusters (Fortunato, 2010). Infomap diverges from the previous approaches by compressing the information obtained from the random walks using a constrained minimization of the conditional information of the original graph given the signal of the compression. This compression assists in computationally efficient clustering for very large graphs. In this way, Infomap arrives at the optimal compression that contains the most information needed to describe the process of information diffusion found in the graph. The minimization is carried out as a combination of simulated annealing combined with an adaptation of the greedy search used in the Louvain method (Blondel et al., 2008; Rosvall and Bergstrom, 2008). Much like the Louvain method described above, the nodes are iteratively joined to arrive at latent supernodes, and adjacencies among these are then considered. This approach may capture flows of information better than modularity, which solely focuses on pairwise relationships. Prior work on simulated sparse count matrices reveal that Infomap may outperform approaches that optimize modularity (Lancichinetti and Fortunato, 2011).

### 2.2. Simulations

**Figure 2:**
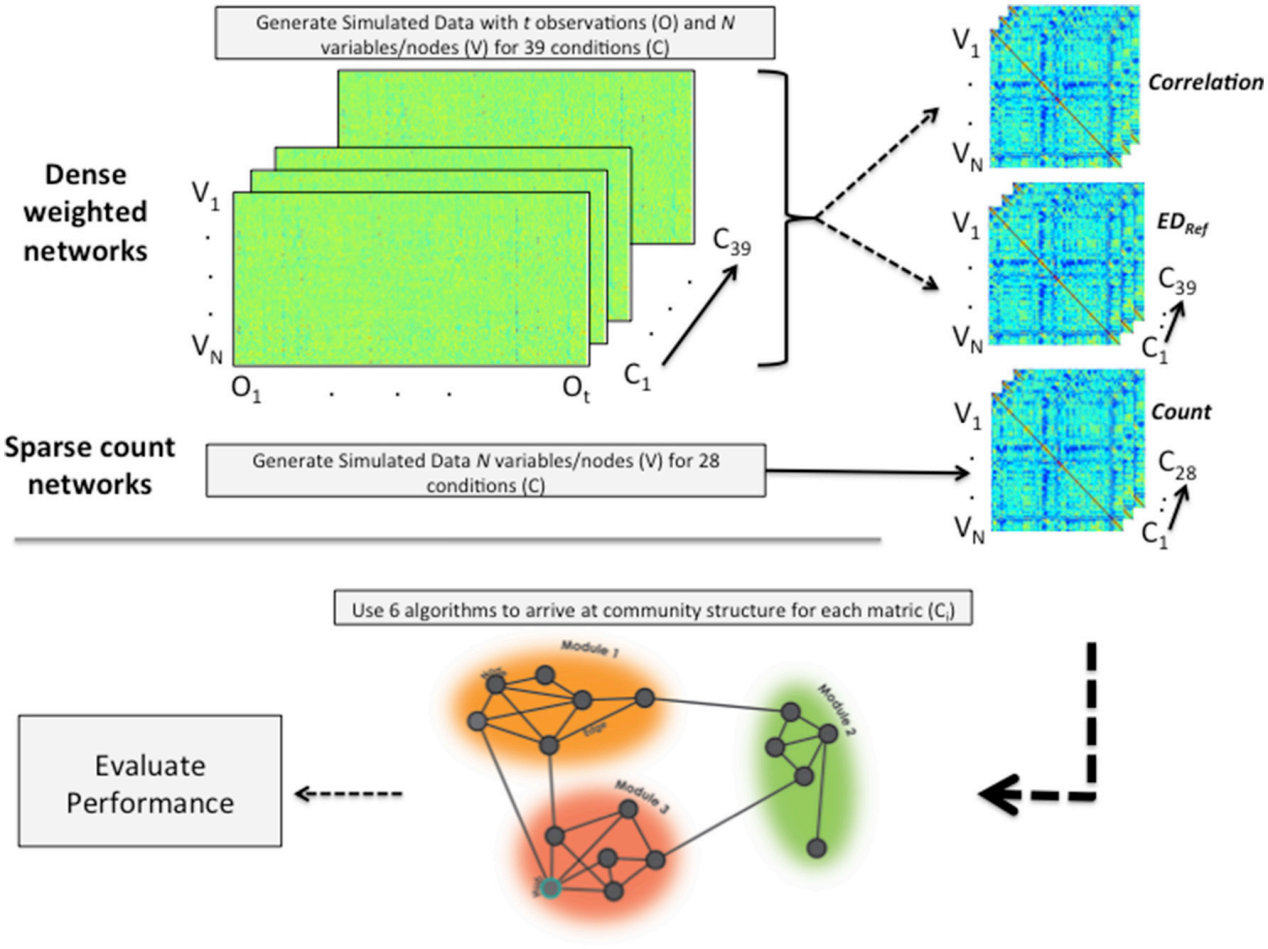
**Depiction of data simulation and analytic process**. For dense weighted networks, time series data are generated. From these, correlation and (reflected) Euclidean distance matrices are computed. Sparse count matrices are made directly using the LFR benchmark simulation program. All data generated across all conditions are then subjected to each of the 6 community detection algorithms and their performance evaluated. “V” indicates variable or node; “O” indicates the observation, and “C” the condition.

**Figure 3:**
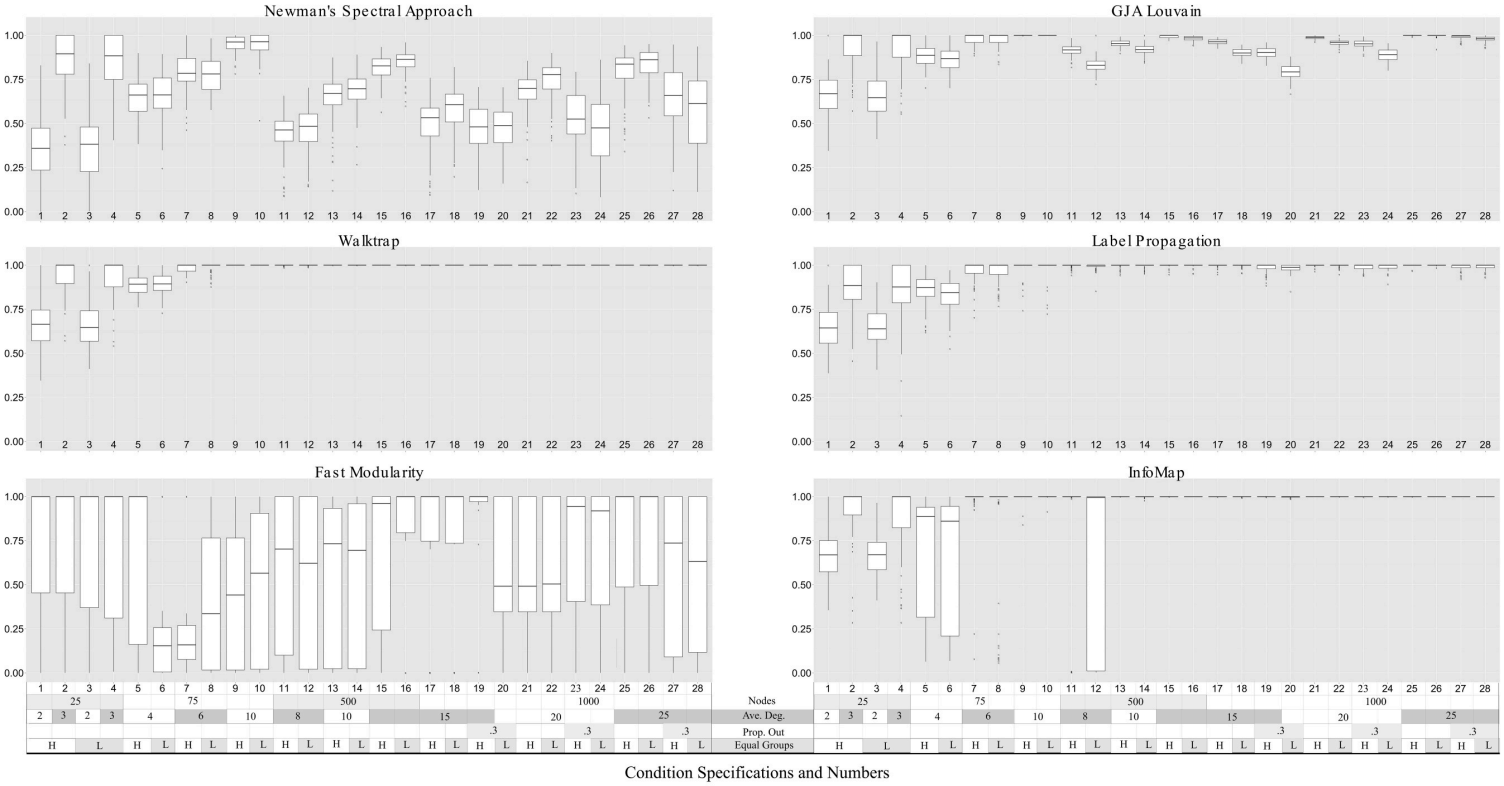
**Community precision for LFR count matrices**. Simulation numbers correspond to those described in Table 2. In the legend depicting the simulation parameters appended at the bottom of the graph, “Ave.” indicates average, “Prop. Out” indicates the proportion of edges that connect nodes with other nodes outside their community (“mixing parameter,” “L,” and “H” indicates low and high number of equally-sized groups (“community distribution exponent”), respectively. No approach could reliably return community structures in count matrices with nodes = 25. Walktrap and Label Propagation consistently performed nearly perfectly for count matrices with nodes = 500 and higher.

**Figure 4:**
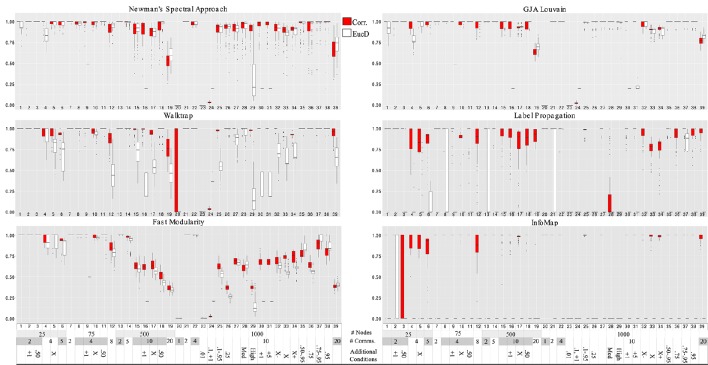
**Hubert-Arabie adjusted Rand Index ARI_*HA*_ for correlation and reflected euclidean distance matrices**. Simulation numbers correspond to those described in Table 3. In the legend depicting the simulation parameters appended at the bottom of the graph, “Comms.” indicates communities; in the “Additional” row, numbers indicate the connectivity range (when not stated, it is 0.25–0.95); “X” indicates unequal group sizes, with “X−” indicating more small groups than large and “X+” indicating more large groups than small; “Med” and “High” indicate medium and high degrees of community overlap (i.e., correlation among communities), respectively. Infomap conducted on correlation matrices performed best for graphs with 25 nodes. Walktrap on correlation matrices outperformed the other methods for graph sizes ≥75 across the varied conditions. Walktrap and Infomap conducted on correlation matrices were the only approaches to not return false communities when there is only 1 community (Simulation #20).

When simulating the data we followed what Radicchi et al. (2004) define as a community in a strong sense. That is, for each node the degree distribution in terms of weights of edges is higher with nodes within its community than with nodes outside its community. The focus of the present investigation on weighted, undirected graphs limited our options in terms of available benchmark-generating programs. Data were thus generated within R using three methods (see Figure 2), two of which were data generation algorithms developed in-house. The first data generation algorithm is the publicly available Lancichinetti-Fortunato (LFR) benchmark approach for arriving at sparse count matrices (Lancichinetti et al., 2008). These count matrices emulate the count of structural connections in the brain. Given the high use of community detection algorithms on correlation matrices obtained from fMRI data, the second approach (developed by the present authors) creates correlation matrices by generating model-implied multivariate time series data. Each of these variables (e.g., brain regions of interest) are similar to other variables in their community in terms of their activity across time and are less similar to those outside their community. The third approach generates Euclidean distance matrices from the data used in the second approach. As the Euclidean distances indicate differences rather than similarities, this measure is reflected. These data simulations enable investigation into the circumstances under which each approach may prevail when a researcher must choose between generating a correlation or a Euclidean distance matrix (a situation common for functional MRI researchers with data for many regions across time). Below we offer brief descriptions of the simulation procedures, with the specific conditions indicated in Tables 2, 3 as well as in the X-axes of Figures 3, 4. Details of the simulations are provided in the Supplemental Information for the interested reader. The full set of simulated networks can be found here: https://gateslab.web.unc.edu/simulated-data/weighted-community-detection/.

**Table 2:** **LFR simulations**.

**Number**	**Nodes**	**Ave. Degree**	**Max Degree**	**Prop. Out**	**Hetero. Size**
1	25	2	4	0.1	1
2	25	3	4	0.1	1
3	25	2	4	0.1	2
4	25	3	4	0.1	2
5	75	4	15	0.1	1
6	75	4	15	0.1	2
7	75	6	15	0.1	1
8	75	6	15	0.1	2
9	75	10	15	0.1	1
10	75	10	15	0.1	2
11	500	8	30	0.1	1
12	500	8	30	0.1	2
13	500	12	30	0.1	1
14	500	12	30	0.1	2
15	500	15	30	0.1	1
16	500	15	30	0.1	2
17	1000	15	50	0.1	1
18	1000	15	50	0.1	2
19	1000	15	50	0.3	1
20	1000	15	50	0.3	2
21	1000	20	50	0.1	1
22	1000	20	50	0.1	2
23	1000	20	50	0.3	1
24	1000	20	50	0.3	2
25	1000	25	50	0.1	1
26	1000	25	50	0.1	2
27	1000	25	50	0.3	1
28	1000	25	50	0.3	2

*“Ave.” indicates “Average”; “Prop. Out” indicates the proportion of edges that nodes' have with nodes outside their community; “Hetero.” refers to “Heterogeneous” with larger values indicating more equality with regards to community sizes*.

**Table 3:** **Correlation and euclidean distance simulations**.

**Number**	**Nodes**	**Conn. Range**	**Hetero. Comm. Size**	**# of Comms**.	**Overlap**	**Level Diff**.
1	25	0.25–0.95	Equal	2	0.1	0
2	25	0.25–0.95	Equal	2	0.1	1
3	25	0.5–0.5	Equal	2	0.1	0
4	25	0.25–0.95	Equal	4	0.1	0
5	25	0.25–0.95	As many large as small	4	0.1	0
6	25	0.25–0.95	Equal	5	0.1	0
7	75	0.25–0.95	Equal	2	0.1	0
8	75	0.25–0.95	Equal	4	0.1	0
9	75	0.25–0.95	Equal	4	0.1	1
10	75	0.25–0.95	As many large as small	4	0.1	0
11	75	0.5–0.5	Equal	4	0.1	0
12	75	0.25–0.95	Equal	8	0.1	0
13	500	0.25–0.95	Equal	2	0.1	0
14	500	0.25–0.95	Equal	5	0.1	0
15	500	0.25–0.95	Equal	10	0.1	0
16	500	0.25–0.95	Equal	10	0.1	1
17	500	0.25–0.95	As many large as small	10	0.1	0
18	500	0.5–0.5	Equal	10	0.1	0
19	500	0.25–0.95	Equal	20	0.1	0
20	1000	0.25–0.95	Equal	1	0.1	0
21	1000	0.25–0.95	Equal	2	0.1	0
22	1000	0.25–0.95	Equal	4	0.1	0
23	1000	0.01–0.01	Equal	10	0.1	0
24	1000	0.1–0.1	Equal	10	0.1	1
25	1000	0.1–0.95	Equal	10	0.1	0
26	1000	0.25–0.25	Equal	10	0.1	0
27	1000	0.25–0.95	Equal	10	0.1	0
28	1000	0.25–0.95	Equal	10	0.25	0
29	1000	0.25–0.95	Equal	10	0.75	0
30	1000	0.25–0.95	Equal	10	0.1	1
31	1000	0.25–0.95	Equal	10	0.1	5
32	1000	0.25–0.95	More large than small	10	0.1	0
33	1000	0.25–0.95	As many large as small	10	0.1	0
34	1000	0.25–0.95	More small than large	10	0.1	0
35	1000	0.5–0.95	Equal	10	0.1	0
36	1000	0.75–0.75	Equal	10	0.1	0
37	1000	0.75–0.95	Equal	10	0.1	0
38	1000	0.95–0.95	Equal	10	0.1	0
39	1000	0.25–0.95	Equal	20	0.1	0

*“Conn. range” refers to the connectivity range for each node connecting with their community with high values indicating high connectivity; “Hetero.Comm. Size” describes degree of heterogeneity in size of communities with communities being either “equal” in size, different sized (large or small) but in the same proportion, or different sized with one size dominating; “# of Comms.” refers to the number of communities; “Overlap” refers to the degree to which communities are related in their connectivity; “Level Diff.” refers to communities having different overall averages of within-network connectivity*.

#### 2.2.1. Simulation of count matrices

Sparse count represent the types of matrices used by researchers examining anatomical connectivity by counting streamlines to arrive at the total number of tracts between two given brain regions (Kaiser, 2011). The matrices become sparse become some brain regions are not structurally connected. Count matrices were generated using the Lancichinetti and Fortunato (LFR) algorithm (Lancichinetti et al., 2008). Graphs produced by the LFR benchmark follow the power law distribution for degree. Power law distribution appropriately describes data that has a long tail containing rare but extreme values (Clauset et al., 2009) and has become a guiding property in the generation and testing of algorithms (Newman, 2006; Barabási, 2013). This distribution has long been considered an aspect inherent in many networks containing counts, from large networks such as the world wide web (Albert et al., 1999) to biological systems (Clauset et al., 2009) to smaller networks of scientific collaborators (Newman, 2001). Brain networks also appear to have similar distributional properties as those seen in these other contexts (Bassett and Bullmore, 2006). While other generative programs for count matrices also follow a power-law distribution, we chose the LFR benchmark program primarily because it allows for variation in node degree average and community size.

The LFR algorithm for generating benchmarks allows the user to define parameters relating to the (1) graph size (number of nodes), (2) average and (3) max degree for each node (drawn from a power-law distribution), (4) the proportion of links that are outside their community vs. in, and (5) heterogeneity in the community sizes. The simulation conditions are specified in Table 2 (also indicated at the 666 bottom legend of Figure 3). In terms of graph size, we tested low numbers of nodes (25 and 75) for which the methods had previously not been tested. The average and max degrees increased as graph size increased given the increased number of nodes and were informed by prior studies (Lancichinetti and Fortunato, 2009). For the majority of conditions, the proportion of connections that are out of the community was low (0.10) but we also tested a higher proportion of out-degree (0.30). Finally, we tested the impact of heterogeneity in the size of communities. One hundred graphs were generated for each condition.

#### 2.2.2. Simulation of correlation and euclidean matrices

Here we offer a conceptual overview with details needed for replication contained in the Supplemental Material. In following the current state of science and use of correlation matrices with community detection (Zuo et al., 2012; Sporns, 2013), we generate matrices which reflect only the lag-0 relations among brain regions. As seen in previous fMRI studies, the correlation matrices contain negative correlations and connection weights that span from near zero to very strong (Fair et al., 2009; Gates et al., 2014). We also generate Euclidean Distance (EucD) matrices since this offers an alternative to correlation matrices that may be preferred if the researcher wishes to retain information regarding the level of the brain activity (i.e., the scale) in addition to the degree to which the series fluctuates across time in accordance with another region (which is the only information captured by correlation).

The generative algorithm for arriving at the full, weighted correlation and Euclidean distance matrices stems from structural equation modeling (SEM). The approach first arrives at a population-level correlation matrix for each replication of each condition indicated in Table 3; from this matrix, multivariate time series data are generated. The specific conditions are set as follows. First, we define the number of nodes in the graph (much like in the LFR count matrix simulations) with graphs as small as 25 nodes. Next, the number of communities is set. We then indicate how distinct the communities are by allowing varying degrees of overlap (i.e., correlation among the communities or latent variables in SEM nomenclature). Fourth, we specify the connectivity range within communities for how strongly they relate to other nodes within the community (set by the factor loading matrix loading on the latent community variable). High numbers here indicate a higher degree of connectivity for that node with the rest of those in their community. Finally, we specify the heterogeneity in community sizes. This platform for data generation is highly flexible, enabling us to dictate directly the number of communities, as well as how much each individual node is connected to its community as well as other communities.

From the joint probability specified by the population-level correlation matrices we generated random variables to arrive at *N*-variate by *t*-observations data matrices for each data replication in each condition. These data matrices have a large number of observations across time (*T*≥ 502 for graph sizes of 25–500; *T*≥ 1002 for graph sizes of 1000). These sizes were chosen such that there are more observations than nodes and also are in line with the number of time points expected in a block or resting state fMRI study. Much like the LFR matrices, these data can be considered to be random pulls from the population, and 100 data matrices for each condition are generated in this manner. At this point, the data produced here is in the same format as functional MRI data collected across time for specific brain regions. From these time series data matrices, both correlation and Euclidean distance matrices are generated using standard equations. The resulting correlation matrices are directly used to evaluate the methods. The Euclidean distance matrices must be further manipulated since the approaches used herein expect similarity matrices and the Euclidean distance matrices are difference matrices with high values indicating low connectivity. The values in the Euclidean distance matrix are reflected by taking the absolute value of the difference between each value and the highest Euclidean distance in the matrix. Thus, what was originally the highest distance becomes zero (for no similarity), and the shortest distance becomes high in value (for high connectedness), resulting in a reflected Euclidean Distance Matrix (EucD).

An important step taken prior to conducting community detection algorithms is setting the diagonal in each correlation and EucD matrix to zero. The diagonal here represents the similarity that a given node has with itself, and thus from a conceptual viewpoint is not informative in arriving at communities. Analytically it may cause problems because the diagonal contains the highest value found in the matrix for both the correlation and EucD matrix, with the diagonal being ones for the former and the highest value in the original Euclidean distance matrix for the latter. While NSA and GJA algorithms would be unaffected by this choice, Walktrap, Fast Modularity, Label Propagation, and Infomap will be affected. For consistency, we present in the main text the results for the analysis conducted with the diagonal set to zero as this provided superior results for Label Propagation while returning similar rates of performance on the aggregate for Walktrap, Fast Modularity, and Infomap.

### 2.3. Evaluation of community detection methods

Three indices are used for evaluating appropriate community designation across the simulations: majority placement, the Hubert-Arabie Adjusted Rand Index, and modularity ratio. “True community” here refers to the community to which a node was assigned in the generating procedures. These measures have been extensively used to describe the accuracy of each community detection method's ability to place nodes in the correct community. Finally, multiple regression analysis was conducted for each community detection method using the Hubert-Arabie Adjusted Rand Index as the dependent variable to identify conditions that influenced appropriate recovery of community structures. *Post hoc* comparisons between methods were conducted to arrive at effect sizes for the differences in their accuracy.

#### 2.3.1. Majority placement

The first index is the commonly used Girvan and Newman (2002)'s approach of looking at the fraction of correctly classified nodes. This is referred to here as *majority placement* (MP) because nodes are placed with the majority of their true community. Here, nodes placed in a cluster with the majority (“>” 50%) of the other nodes in its true community is identified as being in the correct cluster (Fortunato, 2010), providing a number between 0 and 1 which indicates the proportion of nodes with correct community identification. Formally,

(2) $$MP=\overset{N}{\underset{i\;=\;1}{\sum }}\tau_{i}/N,\;\tau_{i}=\{\begin{array}{cc} 1\begin{array}{l} \text{if node }i\text{ placed with}\geq 50\%\\ \text{ of true community} \end{array}\\ 0 & \text{otherwise} \end{array}$$

where for each individual *i*, τ is “1” if the individual is in a community with at least 50% of others in their true community. A benefit of this approach is that it quantifies one of the underlying premises driving community detection: nodes are placed with others with whom they are highly connected. A critical drawback is that the results are biased and tend to overinflate accuracy. For instance, in the case where only one community is identified, every node will be categorized as “correct” placement since they are with the majority of their fellow community members (see Figure 1C for a visual depiction of “Super Community”). Hence it will be inflated for when there are fewer communities found than there are in the generated data (i.e., high sensitivity, but low specificity).

#### 2.3.2. Hubert-arabie adjusted rand index

Classification rates, a category that MP falls into, may not be the best choice because of the arbitrary distinctions necessary to arrive at what the “correct” placement is (Steinley, 2004). Given this insight and the inflated bias sometimes seen in MP, we also used a second metric that better addresses specificity, *Hubert-Arabie Adjusted Rand Index* (ARI_*HA*_; Hubert and Arabie, 1985). ARI_*HA*_ complements MP by assessing the precision of node assignment to the correct community and is a stricter criterion than MP in that it accounts for chance placement of nodes. Formally, the ARI_*HA*_ is defined as:

(3) $$ARI_{HA}=\frac{\left(\begin{array}{c} N\\ 2 \end{array}\right)(a+d)-[(a+b)(a+c)+(c+d)(b+d)]}{{\left(\begin{array}{c} N\\ 2 \end{array}\right)}^{2}-[(a+b)(a+c)+(c+d)(b+d)]}$$

where each pair of nodes provides a count for either *a, b, c*, or *d*. The value *a* indicates the number of pairs placed in the same community for both the true and recovered partition. Both *b* and *c* indicate wrong placement of nodes, with the former indicating the counts of pairs in the same group for the true community structure but different groups for the recovered structure (and the latter indicating the opposite). Finally, *d* indicates the count of pairs that are in different communities in the generated data and also different communities in the recovered structure. The ARI_*HA*_ has an upper limit of 1.0, which indicates perfect recovery of the true community structures and lower values indicating incrementally poorer recovery. The R package *clues* was used to calculate ARI_*HA*_ (Chang et al., 2010). In the case of super communities, ARI_*HA*_ penalizes for the placement of two nodes from different true communities into the same community. In this way, ARI_*HA*_ is tightly linked to the detection algorithms ability to recover the correct number of communities and penalizes for combining two communities into one (i.e., requires high sensitivity and high specificity) or splitting a community into two smaller ones.

#### 2.3.3. Modularity ratio

The third metric is *modularity ratio*, or MR. We utilize MR to evaluate how closely the recovered modularity normed by the true modularity for the generated community structure corresponds with the index of accurate community detection recovery (i.e., ARI_*HA*_). Modularity has been used by a number of studies to demonstrate superiority of methods (e.g., Newman, 2006). Utilizing modularity (*Q*) as a descriptive property of the network, we used Equation (1) to arrive at the modularity in the generating graph clustering assignments, *Q*^*max*^, and the recovered modularity, *Q*^*r*^. The same values are used in both except the δ indicator will differ based on recovered community assignments. The ratio of the recovered *Q*^*r*^ over the maximum *Q*^*max*^ is taken. Thus, we arrive at a proportion of the maximum true modularity in the recovered solution. Modularity values above 0.3 is often considered an indication of good community assignments (Newman, 2006). We wished to investigate the extent to which modularity should be used as an absolute (rather than comparative) indication of appropriate community structure recovery.

#### 2.3.4. Effect sizes of differences

Cohen's *d* was used to quantify the effect sizes for *post hoc* comparisons of mean differences between the methods (Cohen, 1988). Specifically,

(4) $$\begin{array}{l} d=\frac{{\bar{ARI_{HA}}}_{i}-{\bar{ARI_{HA}}}_{j}}{s_{ij}} \end{array}$$

where the average ARI_*HA*_ of a given community detection approach, ${\bar{ARI_{HA}}}_{j}$, is subtracted from the average of one or more other community detection approaches, ${\bar{ARI_{HA}}}_{i}$, for a given condition. The denominator, *s*_*ij*_, indicates the pooled standard deviation. Given the multiple tests possible in this analysis as well as the high power, effects sizes are preferred over significance testing. Conventional interpretations of effects sizes are followed, with the values of 0.20, 0.50, and 0.80 respectively indicating small, medium, and large (Cohen, 1988).

#### 2.3.5. Multiple regression

In order to identify the influence each condition had on the evaluation criteria, a series of multiple regressions were run for each community detection method with the ARI_*HA*_ as the dependent variable. For both the LFR and correlation/EucD matrix simulations, the condition specifiers were dummy-coded with the reference category being the smallest size graph and “typical” situation (e.g., adequate in-degree, or the average strength of within-community connections; low community overlap).

## 3. Results

We first investigated which measures used (MP, ARI_*HA*_, and MR) best related to optimal community recovery. MP was consistently high across most conditions for all methods (see Supplemental Tables 1–3 and Supplemental Figure 1) Thus, it was not useful in discriminating among them. Either all methods performed nearly perfectly across these conditions, or super communities were generated. Super communities (see Figure 1C) occur when multiple communities are subsumed into one large community. MP will still be high in this situation since it does not account for specificity of communities. Hence we do not focus on MP for the majority of the results and only refer to this measure as needed.

We also investigate the utility of using modularity as an indicator for how well-partitioned the modules are. Modularity did not have a clear relationship with accurate community recovery rates. Recall from above that a high modularity ratio of the recovered modularity to the true modularity would suggest that the algorithm found a partition that was as good as the one used to generate the data (see Supplemental Tables 7–9 for full modularity ratio results and Supplemental Figure 2). We see in Table 4 that MR can be unrelated, and in some cases orthogonal, to the actual accuracy of the community structure obtained. For instance, for NSA, Walktrap, and Fast Modularity conducted on the LFR count matrices the effect sizes for the relation between ARI_*HA*_ and MR were moderate to low. A more striking pattern of results was found for NSA, Walktrap, Fast Modularity, and Louvain GJA conducted on correlation matrices, where the effect sizes were 0.00. For, Walktrap connected on the reflected Euclidean matrices, there was actually a negative effect between *ARI*_*HA*_ and MR, suggesting that as accuracy in recovery goes down, MR goes up (and conversely, as MR goes down, the ability to recover the true communities rises). Taken together, these results suggest a low coherence between modularity and identification of the true communities for NSA, Walktrap, Fast Modularity, and Louvain GJA. Thus, while modularity may be useful for identifying the best partition among multiple solutions for the same graph, it may not be the best approach for comparing solutions between graphs.

**Table 4:** **Modularity Ratio as an Indication of Accurate Community Assignments**.

**Method**	**Correlation**	**EucD**	**LFR**
NSA	−0.04	−0.03	0.48
Walktrap	−0.05	−0.30	0.39
Fast modularity	−0.03	0.01	0.41
GJA	−0.05	−0.04	0.68
Label Prop.	0.98	0.99	0.92
Infomap	0.99	1.00	0.89

*Correlation coefficients for relations between ARI_HA_ and Modularity Ratio (MR) across all trials for simulated correlation, EucD, and LFR matrices*.

This incongruence indicates that modularity may not be the best measure of accuracy in community structure recovery for all methods. These issues have been highlighted previously by Fortunato (2010), namely that high modularity does not necessarily indicate that the network has a community structure and that sub-optimal partitions can reach the same modularity value. Obtaining a high modularity may not always reflect that the community structure as defined here was obtained (also see Karrer et al., 2008). Interestingly, Label Propagation and Infomap provided noteworthy exceptions in the present paper with regards to the correlation between accuracy in communities and MR—these two measures are nearly perfectly related for the correlation and EucD matrices, and the effect sizes for LFR matrices were also high. Full results for the modularity ratios across conditions and methods can be found in the Supplemental Information. For reasons outlined here, results presented in the main text concern only ARI_*HA*_.

### 3.1. Sparse count matrices

Walktrap outperformed the other methods in terms of average ARI_*HA*_ across all conditions, with effect sizes for the differences between Walktrap and other methods ranging from small (*d* = 0.14 for Label Propagation) to large (*d* > 1.30 for Fast modularity and NSA; see Supplemental Table 10). This follows previous findings on sparse count matrices that indicated Walktrap performs excellently across a range of conditions (Pons and Latapy, 2006; Orman and Labatut, 2009). Fast Modularity was among the worst performing methods for these sparse count matrices. While exhibiting higher average ARI_*HA*_ than NSA (*d* = 0.42), Fast Modularity exhibited such wide variability in results for each condition (ranging from ARI_*HA*_ = 0.00 to ARI_*HA*_ = 1.00 for many simulations) that it is not recommended for the conditions examined here due to the inconsistency of results. Walktrap's superior performance is consistent with previous findings that simulated sparse matrices (Orman and Labatut, 2009). Indeed, for graphs with 500 or more nodes Walktrap recovered the nodes' appropriate community 100% of the times. This near-perfect performance for large graphs was not the only driver of Walktrap's superior overall performance: for graphs sized 25 and 75, Walktrap's average ARI_*HA*_ was 0.90 (SD = 0.15) with an effect size of 0.32 when compared to the ARI_*HA*_ aggregated across other methods (average ARI_*HA*_ = 0.83, SD = 0.20).

**Figure 5:**
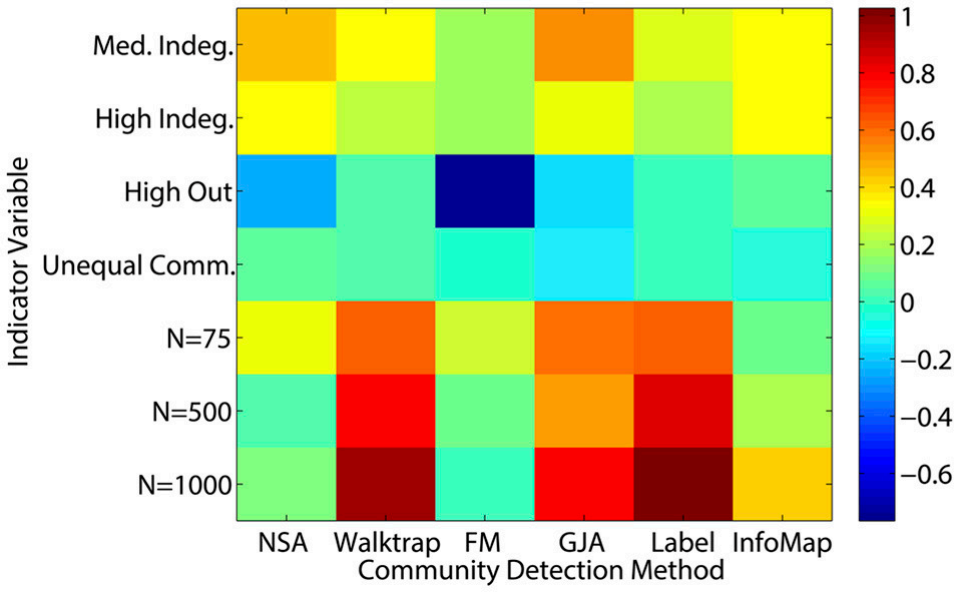
**Heatmap of multiple regression results for LFR count matrices**. Standardized betas are depicted from multiple regressions conducted for each method using the condition indicator variables to predict ARI_*HA*_. “Med” = medium; “Indeg.” = in-degree; “Comm.” = Community; “N” refers to graph size; Reference category is: *N* = 25, equal community sizes, low proportion of outdegree, and low indegree.

A couple of commonalities existed across the methods in terms of the ability to recover the underlying community structure in sparse matrices. None of the methods could appropriately recover communities of size 25 when the in-degree is low (LFR Simulations 1 and 3), with average recovery ranging from ARI_*HA*_ = 0.38 for NSA to approximately ARI_*HA*_ = 0.65 for the other methods (see Figure 3 and Supplemental Table 6 for LFR dataset results). However, overall the regression results for the LFR simulations with ARI_*HA*_ as the dependent variable revealed that unequal community sizes has little effect on any of the methods' ability to recover the true community assignments (see Figure 5).

### 3.2. Correlation and distance matrices

Walktrap conducted on correlation matrices again had overall higher average ARI_*HA*_ than the other methods conducted on either correlation or EucD matrices, with effect sizes ranging from low (*d* = 0.13 for Louvain GJA conducted on correlation matrices) to large (e.g., *d* = 0.71 for Fast Modularity conducted on correlation matrices; *d* ≥ 2.50 for Label Propagation and Infomap conducted on EucD; see Figure 4 and Supplemental Tables 4, 5 and 11). For researchers wishing to use full weighted matrices which may share similar properties to the EucD matrices, Louvain GJA performed the best on these. However, performance was worse than conducting Walktrap on correlation matrices with a small to moderate difference in performance (*d* = 0.37). It is noteworthy that Walktrap performed considerably better for correlation (M ARI_*HA*_ = 0.91, SD = 0.24) than for EucD matrices (M ARI_*HA*_ = 0.74, SD = 0.32) with a medium effect size of *d* = 0.60 for the mean difference.

As noted above, researchers with fMRI data (i.e., numerous temporal observations for many variables) can either generate correlation or reflected Euclidean distance (EucD) matrices that indicate the degree of similarity between two nodes. The decision of whether to generate a correlation vs. an EucD matrix for a given set of data should be informed by the method one wishes to use. For all methods, the EucD matrices returned worse results than using correlation matrices.

Walktrap, Label Propagation, and Infomap, consistently returned the appropriate community structure at rates far higher when using correlation matrices rather than using EucD matrices. Of note, these methods require setting negative correlations between nodes to zero, whereas Louvain GJA utilizes information from negative values. Due to the data generating process, negative correlations occur for nodes that are in different communities at around a rate of 50% of the total correlations of a given simulation set. Thus, thresholding by setting negative values to zero provides Walktrap, Label Propagation, and Infomap with an immediate gain by decreasing the number of potential dyads that can be in the same community. In the end, the most robust approach for arriving at communities of functionally related brain regions using fMRI data is to a create correlation matrix, set the diagonal to zero, and conduct Walktrap. If the researcher would rather conceptualize the data as a pure distance matrix such as the reflected Euclidean Distance, then Louvain GJA is the best option (again, setting the diagonal to zero).

### 3.3. Graph sizes

In general the methods performed better as graph size increased. However, increasing the node size did not relate to improved performance for all methods. For instance, when looking at recovery of communities in sparse count matrices, Newman's Spectral Approach performed worse as seen in the performance for simulations 11 and higher which had graph sizes of 500 and larger (M ARI_*HA*_ = 0.62). Both MP and ARI_*HA*_ followed the same pattern, suggesting that the problem in community recovery for NSA is not super communities but rather the generation of communities comprised of nodes that do not belong together (depicted as“Poor Recovery” in Figure 1).

Small graph sizes of 75 and lower presented problems for most algorithms when looking across all matrix types. Walktrap (M ARI_*HA*_ = 0.90, SD = 0.15), Louvain GJA (average ARI_*HA*_ = 0.89, SD = 0.14), and Label Propagation (M ARI_*HA*_ = 0.89, SD = 0.11) all performed about equally well on the small sparse count matrices. While high relative to the other methods tested here, it must be noted that these performance rates are passable but not exceptional. The majority of methods performed well for small graph sizes on correlation matrices (e.g., Walktrap had a mean ARI_*HA*_ = 0.97, SD = 0.07). This suggests that for small weighted networks, maintaining the full information rather than thresholding to create binary graphs appears to work well.

### 3.4. Ill-defined communities in the generated graphs

We also tested the algorithms under a number of additional conditions that emulate qualities of graphs that would be outside the control of the researcher. These would be conditions such as low within-community connectivity (i.e., low average in-degree), unequally sized subgroups, small subgroups, overlap in communities, and the absence of communities in the graph (i.e., one community). As noted above, Walktrap generally outperformed on average across conditions, and as such typically performed well in these instances.

With regards to the sparse count matrices, Walktrap and Label Propagation were robust even in the presence of relatively high proportion of out- to in-community degree, low average degree, and similar sized communities. Similar to previous findings Fortunato (2010), approaches based from traditional modularity-optimization approaches (i.e., Louvain GJA, Fast Modularity, and Newman's Spectral approach) as well as Infomap performed poorer on the sparse count matrices as average node degree decreased in the count matrices.

For the correlation matrices, as expected none of the community detection methods could recover the true communities for low connectivity ranges (i.e., extremely low within-group average weights of 0.01). This is seen in Simulation 23, where the average ARI_*HA*_ across each method was 0.00. Indeed, while the nodes were placed into communities during data generation, it could be argued that this condition had no communities given the low within-community connectivity.

**Figure 6:**
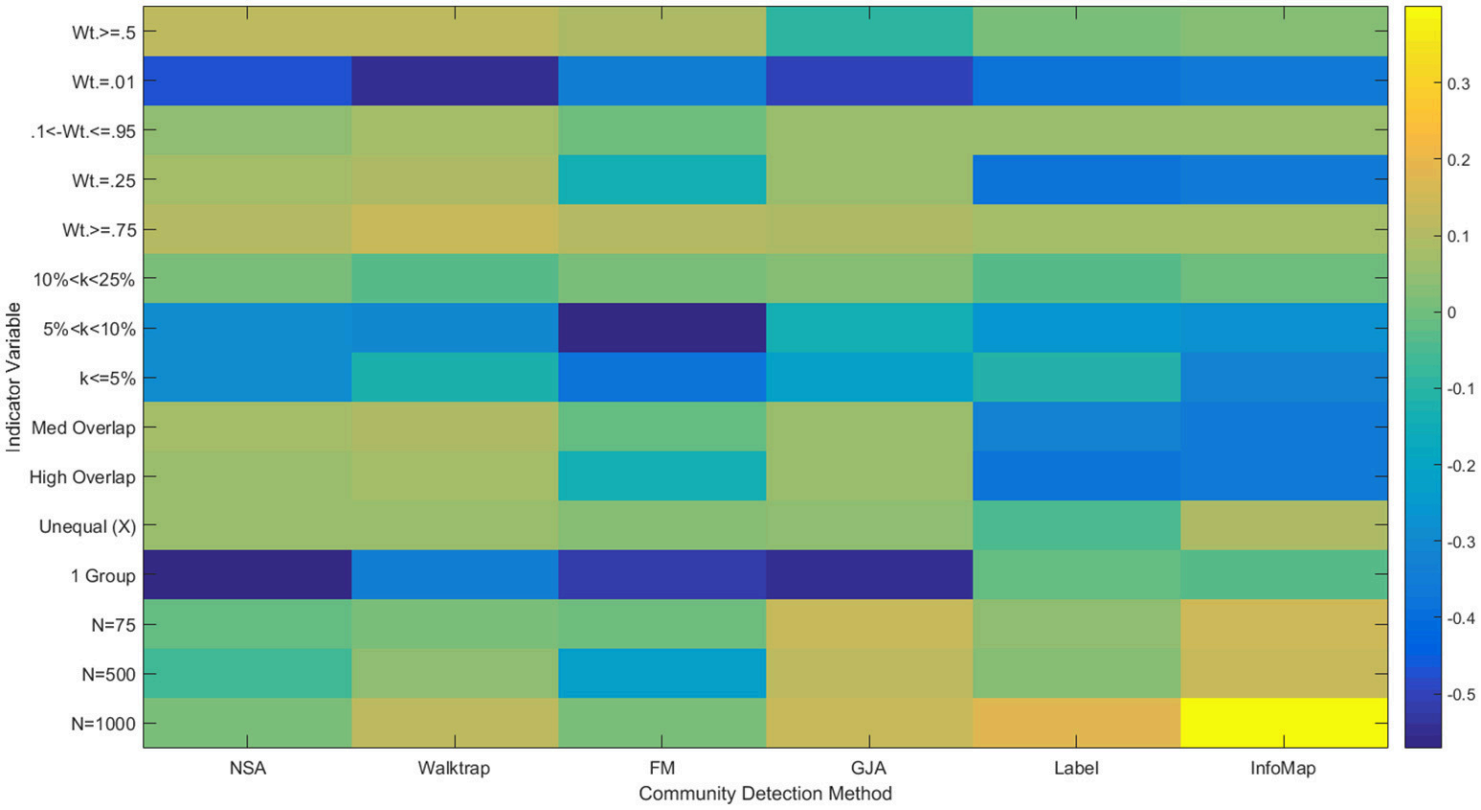
**Heatmap of multiple regression results for correlation matrices**. Standardized betas are depicted from multiple regressions conducted for each method using the condition indicator variables to predict ARIHA. “Wt.” = indegree connectivity weight; “*k*” refers to the proportion of the sample in each community; “Med” = Medium; “Unequal” refers to unequal community sizes; “*N*” refers to graph size. Reference category is: *N* = 25, equally sized communities, low overlap, each community containing at least 25% of the sample, and indegree weight between 0.25 and 0.95.

**Figure 7:**
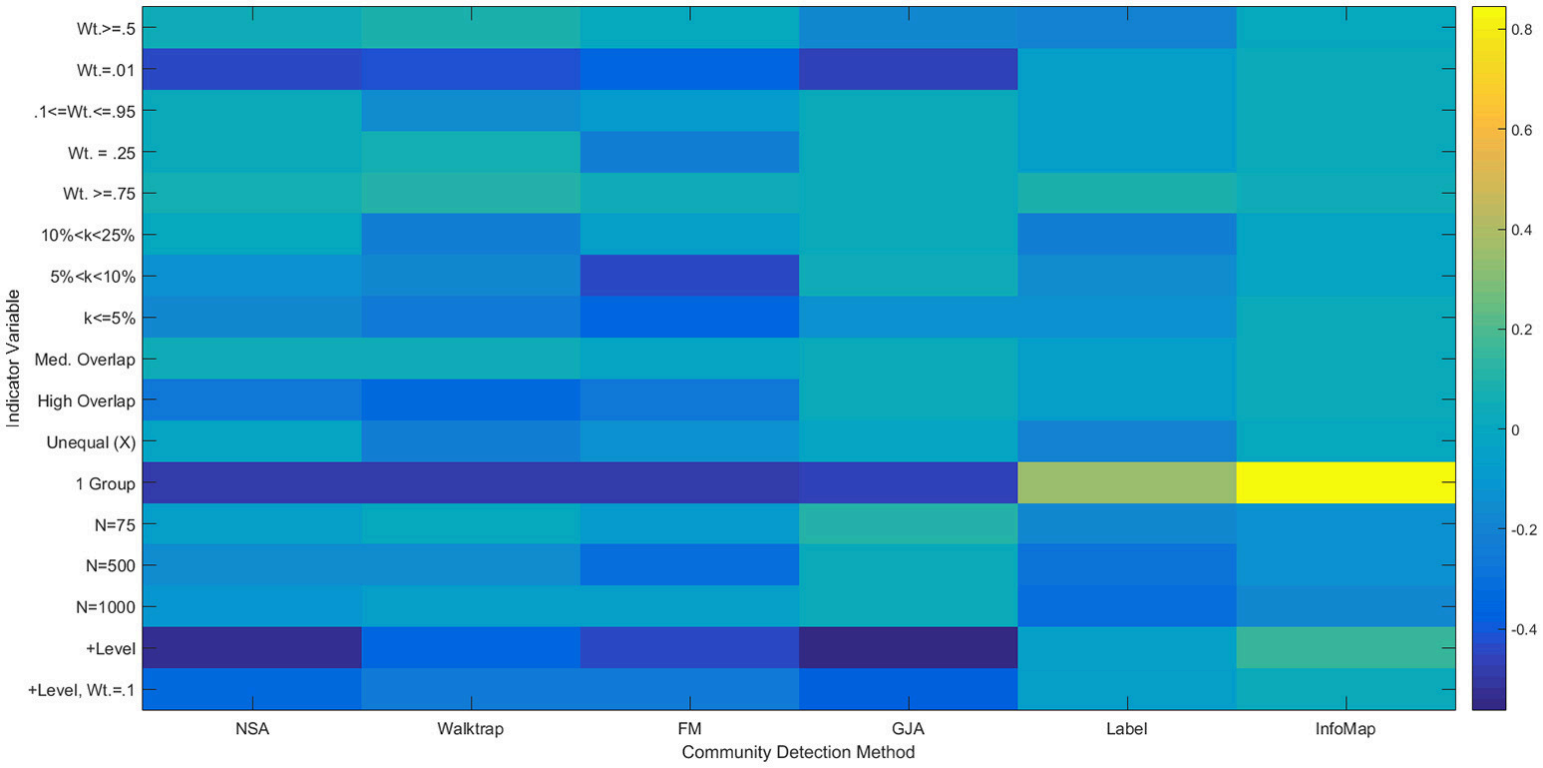
**Heatmap of Multiple Regression Results for Reflected Euclidean Matrices**. Standardized betas are depicted from multiple regressions conducted for each method using the condition indicator variables to predict ARI_*HA*_. “Wt.” = indegree connectivity weight; “k” refers to the proportion of the sample in each community; “Med” = Medium; “Unequal” refers to unequal community sizes; “N” refers to graph size. Reference category is: *N* = 25, equally sized communities, low overlap, each community containing at least 25% of the sample, and indegree weight between 0.25 and 0.95.

Regression results revealed that all of the approaches experienced decreased performance rates when there were a greater number of subgroups containing smaller percentages of the sample. For instance, when tasked with identifying 20 communities with 50 nodes assigned to each (i.e., each subgroup contained 5% of the sample; Simulation 39), ARI_*HA*_ on correlation matrices ranged from an average of 0.59 for NSA to 0.94 for Walktrap and Label Propagation. All methods had high MP for this condition, indicating that super communities were generated since nodes were appropriately placed with the majority of their true community members at the cost of there being poor distinction between communities. While Walktrap is sensitive to small communities with recovery rates decreasing as the resolution of the communities increased (see Figures 6 and 7), this is relative to its near-perfect recovery in other conditions. As seen in Figure 4 and specifically Simulation 39, this slight decrease in accuracy is likely not meaningful. Hence Walktrap uniquely can recover communities a high level of specificity when they are small.

Both Walktrap and Louvain GJA were robust in the presence of poorly defined communities. This includes cases when there is medium to high correlations among the communities as well as conditions with unequally sized communities. In particular, Louvain surpassed most other methods in its ability to recover the underlying groups when correlation among the communities exists: for Simulations 28 and 29, the average ARI_*HA*_ for each was 1.00. This is only slightly higher than Walktrap conducted on correlation matrices (0.99 and 0.98, respectively). Label Propagation was particularly negatively influenced by the presence of overlap in the communities, resulting in an average ARI_*HA*_ of 0.33 (SD = 0.43) for these simulations. Label Propagation and Infomap both performed particularly poorly when the average in-degree was set to 0.25. This result for Label Propagation coincides with results seen in sparse count matrix simulations that found a sharp decline in performance once the in- and out-degree ratio was blurred (Orman and Labatut, 2009). One particularly noteworthy finding is that when all nodes were generated to belong in one community (i.e., there is no community structure) Label Propagation and Infomap were the only approaches that appropriately returned one community. That is, they do not find communities when they do not exist. These were the only methods that accomplished this.

### 3.5. Consistency in solutions for louvain GJA and label propagation

In an auxiliary set of analysis we investigated the degree to which instability in results influenced the findings above. As noted in the description of methods, the Louvain GJA and Label Propagation approaches are non-deterministic. In this way, they may be sensitive to starting points. For this reason researchers sometimes run the algorithms a number of times on the same data set and choose a solution that appears consistent. We followed suit here by running the algorithms 100 times on each data set for each of the 100 matrices within the conditions. We present here the deviation in *ARI*_*HA*_ across these runs (see also Supplemental Tables 12 and 13).

The standard deviations in the appropriateness of solutions for running Louvain GJA on the sparse count matrices or densely weighted matrices were quite low. Rarely was the deviation in *ARI*_*HA*_ > 0.01. This suggests that, for the conditions tested here, Louvain GJA is relatively consistent across runs.

By contrast, Label Propagation demonstrated a high degree of deviation in it's solutions for the correlation matrices, ranging from an average of 0.00 in some simulations to 0.40 for others. Interestingly, the degree of deviation in the accuracy of solutions did not correspond to overall mean *ARI*_*HA*_ (*r* = 0.04). Thus, inconsistencies in solution do not necessary indicate poor recovery in this case. The primary cause for instability in solutions seems to stem from having small communities. For instance, the solutions were the most inconsistent for Simulation 19, which had the largest number of communities to nodes ratio. For sparse count matrices, Label Propagation was fairly consistent across runs with an average of zero deviation in *ARI*_*HA*_ for multiple runs on the same data set. Supplemental Tables 12 and 13 provide results for the amount of deviation for each data set within each condition.

## 4. Discussion

The present work extends previous findings demonstrating that not all community detection algorithms recover the true community structure well across varied types of weighted matrices. We conducted the first evaluation into the performance of weighted community detection algorithms on matrices which these algorithms are often applied yet not always evaluated: sparse count, correlation, and distance matrices. The present findings indicate that some of the commonly used community detection algorithms do not perform well for correlation matrices. Walktrap (Pons and Latapy, 2006) and Louvain GJA (Blondel et al., 2008) conducted on correlation matrices appears to be the best options available for researchers with fMRI data who wish to identify modular communities of brain regions that function together. For sparse count matrices (such as those used for structural brain connections), Label Propagation (Raghavan et al., 2007), and Walktrap perform equally well under varied conditions.

Modularity appears to be a good stopping mechanism but comes with limitations. In many cases where the true communities were not recovered modularity was still high, with the reverse occurring as well. Hence using modularity alone as a criterion for identifying the underlying community structure may not always indicate that the communities truly have greater within-community than between-community connectivity, and it likely is not the appropriate measure on which to decide if a method is performing appropriately. Optimization of modularity has been shown to have a problem with resolution in that it merges smaller communities into larger communities (Lancichinetti et al., 2011). We see this in the present data, with there being a high ratio of recovered to true modularity even when communities were poorly recovered. It is important to note that while Walktrap utilizes modularity to identify the best partition, it does not optimize modularity during partitioning. Perhaps for this reason it performed better when the communities were relatively small.

The reliability of methods varied greatly. Fast Modularity results in particular varied widely within multiple simulations for the sparse count matrices. For condition number 12, for instance, Fast Modularity sometimes perfectly returned the community and other times had recovery rates around zero. Walktrap and label propagation, by contrast, routinely had small variability in terms of their performance. Thus, they reliably returned the same results across the numerous runs within a condition. For the correlation matrices, Walktrap and Infomap consistently provided the same results within runs. However, Infomap sometimes had recovery of zero for all runs within a condition; thus while being reliable, it consistently provided the wrong results. Two methods—Louvain GJA and Label Propagation—are non-deterministic and thus results may vary even when the exact same data set is used. Our analyses revealed that the deviation from one run to another for the same data set was quite small for Louvain, whereas for Label Propagation the difference in solutions across runs for the same data set could be quite large. Taken together, Walktrap would be an optimal algorithm for investigating consistency of networks across time. Increasingly it is becoming apparent that individuals may vary in their functional connectivity across time (Laumann et al., 2015). Thus, it is imperative that methods such as Walktrap, which is both reliable and accurate, are used when investigating brain functioning across time; otherwise changes in the results may be erroneously produced by the algorithm being unreliable.

A few limitations need be noted. What constitutes a community often can be arbitrary since the concept is somewhat ill-defined (Fortunato, 2010). Hence when the present evaluation identifies that the community detection algorithms appropriately recovered the “true” community structures, it is more appropriately said that it recovered the true communities as we conceived them. Perhaps, in the cases where the recovered modularity was high despite adequate recovery as indicated by the Hubert-Arabie Adjusted Rand Index, the communities did exist but simply under a different definition. That an algorithm performs poorly in one case simply highlights the type of bias or structure of communities it is likely to recover. An additional limitation is that this is not an exhaustive analysis of all community detection algorithms described in the literature. The algorithms chosen were based on public availability and ability to handle undirected weighted graphs. Thus, these results do not extend to directed or unweighted counterparts. It is important to remember that these findings are contingent on the specifications that we have selected for our simulations. While we have done our best to provide realistic tests for the methods, our finding that Walktrap performs best for correlation may not be generalizable to all types of data. Additionally, researchers should attend to the qualities of their data and, using the findings regarding the strengths of each approach for varying conditions, utilize this information as well to make their decision.

## Author contributions

KG conceived the study, supervised data simulation and results, and wrote the majority of the text. TH assisted in development of simulation plan, conducted the simulations and results, and wrote supplemental material pertaining to those. DS assisted in the review of previous methods and identification of optimal evaluation index. DF assisted in the development of simulation plan and writing of the manuscript.

## Funding

This work was supported by NIH Grants R21 EB015573-01A1 (NIBIB; KG) and R21 AA022074-02 (NIAAA; DS).

### Conflict of interest statement

The authors declare that the research was conducted in the absence of any commercial or financial relationships that could be construed as a potential conflict of interest.
