# Supplementary material for: A Monte Carlo Evaluation of Weighted Community Detection Algorithms
Source: Front Neuroinform. 2016 Nov 10;10:45. doi: 10.3389/fninf.2016.00045 (PMC5102890; doi:10.3389/fninf.2016.00045)

Supporting Information for "A Monte Carlo evaluation of weighted community detection algorithms"

Contents

Simulations **1**
Simulation of Count Matrices 1
Simulation of Correlation and Euclidean matrices 1

Tables **2**

Figures **14**

Simulations

Simulation of Count Matrices. Count matrices were generated using the Lancichinetti and Fortunato (LFR) algorithm (Lancichinetti & Fortunato, 2009). Graphs produced by the LFR benchmark follow the power law distribution for degree, which has long been considered an aspect inherent in many networks and thus a guiding property in the generation and testing of algorithms (Barabasi, 2013; Newman, 2006). Many systems, from large networks such as the world wide web (Albert, Jeong, and Barabasi, 1999) to smaller networks of scientific collaborators (Newman ,2001), exhibit power law distribution of edges across the nodes. This property has also been seen in biological systems (Clauset, Shalizi, & Newman, 2007). While other generative programs for count matrices also follow a power-law distribution, we chose the Lancichinetti and Fortunato (2009) benchmark program primarily because it allows for variation in node degree distribution and community size.

Specifically, the Lancichinetti and Fortunato algorithm for generating benchmarks allows the user to define parameters relating to the (1) average degree for each node (k), (2) the proportion of links that are with those in their community versus out (μ_1), (3) the exponent for the power-law distribution of community size (t_2), and (4) the exponent for the weight distribution β. Degrees for each node are drawn from a power-law distribution with the average degree (k), max degree, and exponent (t_1) set by the researcher. To adjust the proportion of in- versus out-degree, the researcher can set the mixing parameter μ_1 which indicates the proportion of a node's links that are connected to nodes outside of its community, additionally researchers can set μ_2 determining what proportion of link weight connects within community vs out of community. Finally, the exponent t_2 dictates the size of the communities with larger values indicating more equality with regards to the community sizes.

Simulation of Correlation and Euclidean matrices. The generative algorithm for arriving at the full, weighted correlation and Euclidean distance matrices stems from measurement theory and is drawn from structural equation modeling. The population correlation matrix Σ, from which the sample correlation matrices are generated, is specified as follows:

$$\Sigma = \Lambda\Psi\Lambda' + \Theta. \quad (1)$$

Here, Λ is an $n \times k$ loading matrix that indicates each node's weight on each community (k), with a minimum of 0 if the node is not assigned to that community and a range of .1 to .95 if it is assigned (see explanation below). The matrix Ψ contains 1s along the diagonal with the off-diagonal elements indicating the correlations among latent factors. Θ indicates the error variance for each node along the diagonal and the correlation of errors (set to zero here)on the off-diagonal elements. In order to return a correlation matrix, $\Theta(i, i)$ is set to $1 - \Lambda_{ik}^2$ since the variance of each node i is known to be Λ_{ik}^2 under the current model.

Λ enables control of a number of factors of interest to the present investigation. Ideal approaches will be able to assess when no real communities exist (Newman, 2006), so we set the lowest number of communities to be one (i.e., all nodes in a graph load on the same factor). To test the ability to find small communities, we have some conditions with more small communities than large, and for each graph size have at least one condition with a large number of communities present.

The second set of conditions discussed in the introduction, that of having the nodes vary in the strength to which they are connected to others in the community, can be addressed in the Λ matrix. This is termed the "connectivity range", with high values indicating stronger connections with the latent community and lower values indicating weaker connections. Values are drawn from this range from a uniform distribution.

The third set of conditions, heterogeneity in community size, is also manipulated with the Λ matrix. Here, we dictate how many nodes belong in each community by assigning loading patterns. A community that is large will have a disproportionate number of individual nodes load on the community, whereas small communities will have more zero loadings than the other communities. In some simulations, there are more small communities than large ones, and in others there is an equal spread of large and small communities and yet others have more large communities than small ones. The majority of the simulations have as equal of community sizes as possible given the divisor of number of communities to the number of nodes. The fourth set of conditions, number of communities, is precisely indicated by setting the number of factors (i.e., columns) in the Λ matrix, with each column corresponding to a community.

To generate node-level data, we consider each node i as a random variable of length 502 observations (except for the 1000 node case, which is 1002), and generate random, multivariate normal variables from the population-derived correlation matrix Sigma. At this point, we can add systematic level offset to individual nodes who belong to the same community k. In this way the conditions for level offset are manipulated. The variables in X are then used to make correlation and Euclidean distance matrices. Note that this parametrization leads to the individual errors being uncorrelated and for each node to be a member of one community. These specifications for each simulation are listed in Table 3 of the main text.

Tables

Supplemental Table 1. Mean Majority Placement (Standard Deviation) for Correlations. Note: LP is Label Propagation, NSA is Newman's Spectral Approach

Condition	NSA	Walktrap	Fast Modularity	Louvain GJA	LP	Infomap
1	1 (0)	1 (0)	1 (0)	1 (0)	1 (0)	1 (0)
2	1 (0)	1 (0)	1 (0)	1 (0)	1 (0)	1 (0)
3	1 (0)	1 (0)	1 (0)	1 (0)	1 (0)	1 (0)
4	1 (0)	0.998 (0.01)	1 (0)	.995 (0.011)	1 (0.004)	1 (0)
5	0.927 (0)	0.997 (0.013)	1 (0)	0.927 (0.044)	0.99 (0.024)	1 (0)
6	0.839 (0)	0.999 (0.007)	1 (0)	0.833 (0.061)	0.997 (0.01)	1 (0)
7	0.944 (0.01)	1 (0)	1 (0)	0.941 (0.012)	1 (0)	1 (0)
8	0.985 (0.017)	1 (0)	1 (0.001)	1 (0)	1 (0)	1 (0)
9	0.784 (0.035)	0.999 (0.004)	1 (0.002)	0.789 (0.035)	0.999 (0.004)	1 (0)
10	0.85 (0.064)	0.991 (0.022)	0.997 (0.012)	0.851 (0.041)	0.994 (0.018)	0.998 (0.011)
11	0.883 (0.025)	1 (0)	1 (0.001)	0.901 (0.01)	1 (0)	1 (0)
12	0.815 (0.038)	0.99 (0.015)	0.998 (0.006)	0.835 (0.032)	0.992 (0.01)	0.999 (0.003)
13	1 (0)	1 (0)	1 (0)	1 (0)	1 (0)	1 (0)
14	0.993 (0.013)	1 (0)	0.99 (0.006)	1 (0)	1 (0)	1 (0)
15	0.976 (0.02)	0.998 (0.003)	0.969 (0.011)	1 (0)	0.999 (0.002)	1 (0)
16	0.984 (0.016)	0.998 (0.004)	0.963 (0.013)	1 (0)	0.998 (0.006)	1 (0)
17	0.952 (0.028)	0.994 (0.009)	0.973 (0.015)	0.993 (0.013)	0.991 (0.01)	0.998 (0.007)
18	0.997 (0.006)	1 (0)	0.986 (0.007)	1 (0)	1 (0)	1 (0)
19	0.971 (0.01)	0.988 (0.008)	0.958 (0.013)	1 (0)	0.999 (0.003)	1 (0)
20	0 (0)	0.48 (0.502)	0.668 (0.023)	0 (0)	1 (0)	1 (0)
21	1 (0)	1 (0)	1 (0)	1 (0)	1 (0)	1 (0)
22	1 (0)	1 (0)	0.999 (0.001)	1 (0)	1 (0)	1 (0)
23	0 (0)	0 (0)	0.206 (0.259)	0 (0)	1 (0)	1 (0)
24	0.432 (0.248)	0.381 (0.272)	0.586 (0.032)	0.073 (0.19)	1 (0)	1 (0)
25	0.986 (0)	0.989 (0.008)	0.939 (0.015)	0.999 (0)	0.997 (0.002)	0.998 (0.002)
26	0.968 (0)	1 (0)	0.953 (0.014)	1 (0)	1 (0)	1 (0)
27	0.998 (0)	1 (0)	0.972 (0.01)	1 (0)	1 (0)	1 (0)
28	0.999 (0)	1 (0)	0.968 (0.01)	1 (0)	1 (0.001)	1 (0)
29	0.974 (0)	0.991 (0.005)	0.92 (0.019)	0.998 (0)	1 (0)	1 (0)
30	0.987 (0.015)	1 (0)	0.973 (0.009)	1 (0)	1 (0)	1 (0)
31	0.986 (0.016)	1 (0)	0.974 (0.01)	1 (0)	1 (0)	1 (0)
32	0.929 (0)	1 (0.001)	0.977 (0.008)	1 (0)	1 (0.001)	1 (0)
33	0.981 (0)	0.997 (0.005)	0.993 (0.005)	0.996 (0)	0.99 (0.012)	0.997 (0.004)
34	0.964 (0)	0.996 (0.006)	0.99 (0.01)	0.98 (0.015)	0.979 (0.018)	0.984 (0.015)
35	0.985 (0)	1 (0)	0.995 (0.003)	1 (0)	1 (0)	1 (0)
36	0.994 (0)	1 (0)	0.992 (0.004)	1 (0)	1 (0)	1 (0)
37	0.998 (0)	1 (0)	0.999 (0.001)	1 (0)	1 (0)	1 (0)
38	1 (0)	1 (0)	0.999 (0.001)	1 (0)	1 (0)	1 (0)
39	0.994 (0)	1 (0.001)	0.965 (0.009)	1 (0)	1 (0.001)	1 (0)

Supplemental Table 2. Mean Majority Placement (Standard Deviation) for reflected Euclidean Distance (EucD) matrices. Note: LP is Label Propagation, NSA is Newman's Spectral Approach

Condition	NSA	Walktrap	Fast Modularity	Louvain GJA	LP	Infomap
1	0.967 (0.035)	1 (0)	1 (0)	0.955 (0.038)	1 (0)	1 (0)
2	1 (0)	1 (0)	1 (0)	1 (0)	1 (0)	1 (0)
3	1 (0)	1 (0)	1 (0)	1 (0)	1 (0)	1 (0)
4	0.952 (0.036)	0.993 (0.02)	1 (0)	0.938 (0.023)	1 (0.005)	1 (0)
5	0.974 (0.031)	0.973 (0.033)	0.998 (0.011)	0.99 (0.017)	0.998 (0.01)	1 (0)
6	0.986 (0.022)	0.99 (0.019)	1 (0)	0.995 (0.012)	0.998 (0.006)	1 (0)
7	1 (0.001)	1 (0)	1 (0)	1 (0.002)	1 (0)	1 (0)
8	0.978 (0.02)	0.994 (0.012)	0.999 (0.003)	1 (0.001)	1 (0.004)	1 (0)
9	1 (0)	1 (0)	1 (0)	1 (0)	1 (0)	1 (0)
10	0.989 (0.024)	0.994 (0.016)	0.994 (0.018)	0.999 (0.002)	1 (0)	1 (0)
11	0.997 (0.008)	1 (0.004)	0.999 (0.004)	1 (0)	1 (0)	1 (0)
12	0.969 (0.022)	0.965 (0.037)	0.995 (0.009)	1 (0.002)	1 (0)	1 (0)
13	1 (0)	1 (0)	1 (0)	1 (0)	1 (0)	1 (0)
14	0.991 (0.017)	0.999 (0.007)	0.982 (0.008)	1 (0)	1 (0)	1 (0)
15	0.967 (0.024)	0.974 (0.029)	0.956 (0.014)	1 (0)	1 (0)	1 (0)
16	1 (0)	1 (0)	1 (0)	1 (0)	1 (0)	1 (0)
17	0.948 (0.028)	0.946 (0.043)	0.959 (0.018)	0.997 (0.007)	1 (0)	1 (0)
18	0.998 (0.003)	1 (0)	0.981 (0.009)	1 (0)	1 (0)	1 (0)
19	0.968 (0.012)	0.913 (0.031)	0.938 (0.016)	1 (0)	1 (0)	1 (0)
20	0.579 (0.007)	0.323 (0.29)	0.583 (0.008)	0.546 (0.009)	1 (0)	1 (0)
21	1 (0)	1 (0)	1 (0)	1 (0)	1 (0)	1 (0)
22	1 (0)	1 (0)	0.999 (0.002)	1 (0)	1 (0)	1 (0)
23	0 (0)	0.6 (0.492)	0.537 (0.01)	0 (0)	1 (0)	1 (0)
24	1 (0)	1 (0)	1 (0)	1 (0)	1 (0)	1 (0)
25	0.984 (0)	0.828 (0.052)	0.897 (0.027)	0.999 (0)	1 (0)	1 (0)
26	0.957 (0)	0.999 (0.001)	0.937 (0.022)	1 (0)	1 (0)	1 (0)
27	0.953 (0)	0.984 (0.024)	0.958 (0.012)	1 (0)	1 (0)	1 (0)
28	0.999 (0)	0.999 (0.003)	0.961 (0.013)	1 (0)	1 (0)	1 (0)
29	0.974 (0)	0.638 (0.074)	0.714 (0.091)	0.998 (0)	1 (0)	1 (0)
30	1 (0)	1 (0)	1 (0)	1 (0)	1 (0)	1 (0)
31	1 (0)	1 (0)	1 (0)	1 (0)	1 (0)	1 (0)
32	0.936 (0)	0.984 (0.025)	0.962 (0.012)	1 (0)	1 (0)	1 (0)
33	0.996 (0)	0.996 (0.01)	0.988 (0.007)	1 (0)	1 (0)	1 (0)
34	0.89 (0)	0.995 (0.01)	0.989 (0.007)	0.973 (0.009)	1 (0)	1 (0)
35	0.991 (0)	1 (0)	0.994 (0.003)	1 (0)	1 (0)	1 (0)
36	0.996 (0)	1 (0)	0.99 (0.005)	1 (0)	1 (0)	1 (0)
37	1 (0)	1 (0)	1 (0.001)	1 (0)	1 (0)	1 (0)
38	0.997 (0)	1 (0)	0.999 (0.001)	1 (0)	1 (0)	1 (0)
39	0.961 (0)	0.97 (0.021)	0.947 (0.012)	1 (0)	1 (0)	1 (0)

Supplemental Table 3. Mean Majority Placement (Standard Deviation) for LFR sparse count matrices. Note: LP is Label Propagation, NSA is Newman’s Spectral Approach

Condition	NSA	Walktrap	Fast Modularity	Louvain GJA	LP	Infomap
1	0.823 (0.08)	0.868 (0.058)	0.868 (0.057)	0.868 (0.057)	0.858 (0.065)	0.861 (0.061)
2	0.953 (0.058)	0.981 (0.041)	0.974 (0.04)	0.981 (0.04)	0.945 (0.076)	0.979 (0.045)
3	0.814 (0.082)	0.859 (0.07)	0.86 (0.07)	0.859 (0.071)	0.85 (0.066)	0.856 (0.068)
4	0.945 (0.07)	0.979 (0.052)	0.966 (0.055)	0.98 (0.048)	0.95 (0.065)	0.98 (0.05)
5	0.835 (0.066)	0.96 (0.028)	0.96 (0.027)	0.973 (0.021)	0.949 (0.03)	0.973 (0.025)
6	0.855 (0.056)	0.963 (0.029)	0.972 (0.021)	0.982 (0.016)	0.953 (0.03)	0.985 (0.019)
7	0.872 (0.062)	0.995 (0.013)	0.986 (0.013)	0.998 (0.007)	0.991 (0.016)	0.996 (0.012)
8	0.878 (0.063)	0.998 (0.007)	0.99 (0.013)	0.999 (0.005)	0.993 (0.017)	0.999 (0.005)
9	0.965 (0.036)	1 (0)	0.992 (0.012)	1 (0)	1 (0)	1 (0)
10	0.961 (0.042)	1 (0)	0.994 (0.01)	1 (0)	1 (0)	1 (0)
11	0.723 (0.032)	1 (0.001)	0.993 (0.003)	1 (0)	0.999 (0.003)	1 (0)
12	0.733 (0.036)	1 (0.001)	0.997 (0.002)	1 (0)	1 (0.001)	1 (0)
13	0.775 (0.052)	1 (0)	0.995 (0.003)	1 (0)	1 (0.002)	1 (0)
14	0.806 (0.055)	1 (0)	0.998 (0.002)	1 (0)	1 (0)	1 (0)
15	0.868 (0.037)	1 (0)	0.996 (0.003)	1 (0)	1 (0)	1 (0)
16	0.9 (0.041)	1 (0)	0.998 (0.002)	1 (0)	1 (0)	1 (0)
17	0.699 (0.032)	1 (0)	0.995 (0.002)	1 (0)	1 (0)	1 (0)
18	0.733 (0.052)	1 (0)	0.998 (0.001)	1 (0)	1 (0)	1 (0)
19	0.808 (0.038)	1 (0)	0.971 (0.006)	1 (0)	1 (0.002)	1 (0)
20	0.829 (0.042)	1 (0)	0.981 (0.005)	1 (0)	1 (0)	1 (0)
21	0.773 (0.052)	1 (0)	0.995 (0.002)	1 (0)	1 (0)	1 (0)
22	0.825 (0.065)	1 (0)	0.998 (0.001)	1 (0)	1 (0)	1 (0)
23	0.861 (0.038)	1 (0)	0.973 (0.006)	1 (0)	1 (0)	1 (0)
24	0.878 (0.032)	1 (0)	0.977 (0.005)	1 (0)	1 (0)	1 (0)
25	0.981 (0.046)	0.981 (0.046)	0.982 (0.043)	0.981 (0.046)	0.982 (0.046)	0.981 (0.049)
26	0.997 (0.002)	1 (0)	1 (0)	1 (0)	0.908 (0.032)	1 (0)
27	0.974 (0.005)	1 (0)	1 (0)	1 (0)	0.918 (0.025)	1 (0)
28	0.976 (0.006)	1 (0)	1 (0)	1 (0)	0.924 (0.024)	1 (0)

Supplemental Table 4. Mean Hubert-Arabie Adjusted Rand Index (ARI_{HA}) (Standard Deviation) for Correlation matrices. Note: LP is Label Propagation, NSA is Newman's Spectral Approach

Condition	NSA	Walktrap	Fast Modularity	Louvain GJA	LP	Infomap
1	1 (0)	1 (0)	1 (0)	1 (0)	1 (0)	0.82 (0.386)
2	0.990 (0.030)	1 (0)	1 (0)	0.984 (0.035)	1 (0)	0.88 (0.327)
3	0.442 (0.097)	1 (0)	1 (0)	0.371 (0.079)	0.78 (0.416)	0.53 (0.502)
4	0.981 (0.045)	0.95 (0.068)	0.954 (0.066)	0.975 (0.043)	0.857 (0.199)	0.859 (0.25)
5	0.987 (0.022)	0.951 (0.085)	0.961 (0.074)	0.999 (0.007)	0.852 (0.159)	0.882 (0.261)
6	0.981 (0.028)	0.902 (0.097)	0.915 (0.07)	0.983 (0.024)	0.838 (0.185)	0.816 (0.255)
7	1 (0)	1 (0)	1 (0)	1 (0.003)	1 (0)	0.95 (0.219)
8	0.978 (0.025)	1 (0)	1 (0.004)	1 (0)	0.997 (0.027)	1 (0)
9	0.987 (0.034)	0.996 (0.024)	0.999 (0.005)	1 (0)	0.961 (0.097)	1 (0)
10	0.978 (0.025)	0.968 (0.034)	0.967 (0.027)	0.982 (0.022)	0.887 (0.089)	0.964 (0.124)
11	0.998 (0.006)	1 (0)	1 (0.004)	1 (0)	0.93 (0.158)	1 (0)
12	0.908 (0.069)	0.872 (0.096)	0.856 (0.071)	0.954 (0.056)	0.838 (0.232)	0.803 (0.259)
13	1 (0.001)	1 (0.001)	1 (0.001)	1 (0.001)	0.99 (0.1)	0.92 (0.273)
14	0.991 (0.015)	1 (0.001)	0.968 (0.034)	1 (0)	0.989 (0.048)	1 (0)
15	0.888 (0.115)	0.98 (0.041)	0.623 (0.054)	0.955 (0.053)	0.941 (0.126)	0.973 (0.12)
16	0.895 (0.122)	0.981 (0.044)	0.617 (0.052)	0.962 (0.053)	0.901 (0.205)	0.97 (0.11)
17	0.856 (0.103)	0.943 (0.048)	0.634 (0.064)	0.925 (0.044)	0.802 (0.257)	0.975 (0.029)
18	0.930 (0.107)	0.987 (0.038)	0.523 (0.057)	0.962 (0.061)	0.785 (0.302)	0.893 (0.286)
19	0.531 (0.100)	0.766 (0.129)	0.361 (0.036)	0.641 (0.049)	0.662 (0.429)	0.009 (0.089)
20	0 (0)	0.48 (0.502)	0 (0)	0 (0)	1 (0)	1 (0)
21	1 (0)	1 (0)	1 (0)	1 (0)	0.99 (0.1)	1 (0)
22	0.973 (0.022)	1 (0)	0.998 (0.002)	1 (0)	0.994 (0.039)	1 (0)
23	0.001 (0)	0 (0.001)	0 (0.001)	0 (0.001)	0 (0)	0 (0)
24	0.034 (0.011)	0.034 (0.011)	0.018 (0.008)	0.028 (0.011)	0 (0)	0 (0)
25	0.898 (0.095)	0.975 (0.02)	0.618 (0.048)	0.988 (0.036)	0.97 (0.105)	0.995 (0.004)
26	0.926 (0.071)	0.999 (0.009)	0.366 (0.034)	0.987 (0.035)	0 (0)	0 (0)
27	0.911 (0.086)	0.996 (0.021)	0.686 (0.054)	1 (0)	0.98 (0.06)	1 (0)
28	0.938 (0.091)	0.989 (0.028)	0.602 (0.048)	1 (0)	0.13 (0.315)	0 (0)
29	0.901 (0.093)	0.975 (0.022)	0.367 (0.03)	0.996 (0)	0 (0)	0 (0)
30	0.948 (0.084)	1 (0)	0.676 (0.046)	0.999 (0.01)	0.979 (0.052)	1 (0)
31	0.954 (0.081)	1 (0)	0.676 (0.049)	0.999 (0.009)	0.987 (0.04)	1 (0)
32	0.917 (0.072)	0.989 (0.023)	0.694 (0.055)	0.944 (0)	0.959 (0.079)	0.989 (0.1)
33	0.878 (0.099)	0.914 (0.031)	0.72 (0.054)	0.904 (0.031)	0.771 (0.086)	0.985 (0.029)
34	0.901 (0.065)	0.92 (0.047)	0.735 (0.071)	0.911 (0.037)	0.767 (0.144)	0.982 (0.016)
35	0.961 (0.082)	1 (0)	0.769 (0.059)	0.997 (0.016)	0.984 (0.038)	1 (0)
36	0.913 (0.150)	1 (0)	0.635 (0.043)	0.997 (0.016)	0.928 (0.137)	1 (0)
37	0.980 (0.086)	0.997 (0.019)	0.895 (0.077)	.998 (0.012)	0.984 (0.047)	1 (0)
38	0.961 (0.115)	0.995 (0.025)	0.802 (0.07)	0.994 (0.023)	0.962 (0.081)	1 (0)
39	0.661 (0.142)	0.941 (0.068)	0.382 (0.03)	0.769 (0.051)	0.946 (0.147)	0.852 (0.333)

Supplemental Table 5. Mean Hubert-Arabie Adjusted Rand Index (ARI_{HA}) (Standard Deviation) for Reflected Euclidean distance (EucD) matrices. Note: LP is Label Propagation, NSA is Newman's Spectral Approach

Condition	NSA	Walktrap	Fast Modularity	Louvain GJA	LP	Infomap
1	1 (0.064)	1 (0)	1 (0)	0.917 (0.069)	0.72 (0.451)	0 (0)
2	1 (0)	1 (0)	1 (0)	1 (0)	0.98 (0.141)	0.4 (0.492)
3	1 (0)	1 (0)	1 (0)	1 (0)	0.01 (0.1)	0 (0)
4	0.847 (0.097)	0.872 (0.101)	0.917 (0.077)	0.787 (0.07)	0.041 (0.154)	0 (0)
5	0.97 (0.04)	0.804 (0.132)	0.918 (0.089)	0.979 (0.035)	0.088 (0.222)	0 (0)
6	0.974 (0.033)	0.683 (0.203)	0.839 (0.112)	0.985 (0.032)	0.06 (0.13)	0 (0)
7	1 (0.003)	1 (0)	1 (0)	0.999 (0.005)	0.65 (0.479)	0 (0)
8	0.97 (0.041)	0.985 (0.037)	0.999 (0.007)	1 (0.002)	0.166 (0.371)	0 (0)
9	0.492 (0)	1 (0)	0.492 (0)	0.492 (0)	0 (0)	0 (0)
10	0.989 (0.02)	0.932 (0.043)	0.962 (0.026)	0.990 (0.019)	0.083 (0.225)	0 (0)
11	0.997 (0.01)	0.997 (0.025)	0.993 (0.038)	1 (0)	0.008 (0.077)	0 (0)
12	0.949 (0.047)	0.458 (0.167)	0.779 (0.078)	0.990 (0.029)	0 (0)	0 (0)
13	1 (0.002)	1 (0)	1 (0.002)	1 (0.001)	0.27 (0.446)	0 (0)
14	0.987 (0.02)	0.984 (0.053)	0.928 (0.067)	1 (0)	0 (0)	0 (0)
15	0.904 (0.106)	0.726 (0.146)	0.593 (0.064)	0.982 (0.038)	0 (0)	0 (0)
16	0.200 (0)	0.305 (0.143)	0.2 (0)	0.2 (0)	0 (0)	0 (0)
17	0.847 (0.11)	0.544 (0.129)	0.566 (0.055)	0.936 (0.042)	0 (0)	0 (0)
18	0.955 (0.098)	0.974 (0.06)	0.419 (0.043)	0.984 (0.039)	0 (0)	0 (0)
19	0.599 (0.095)	0.473 (0.136)	0.337 (0.035)	0.699 (0.05)	0 (0)	0 (0)
20	0 (0)	0 (0)	0 (0)	0 (0)	1 (0)	1 (0)
21	1 (0)	1 (0)	1 (0)	1 (0)	0.53 (0.502)	0 (0)
22	0.973 (0.022)	1 (0)	0.996 (0.004)	1 (0)	0.09 (0.288)	0 (0)
23	-0.001 (0)	0 (0)	0 (0.001)	0 (0.001)	0 (0)	0 (0)
24	0.201 (0)	0.363 (0.021)	0.201 (0)	0.201 (0)	0 (0)	0 (0)
25	0.897 (0.091)	0.551 (0.097)	0.519 (0.053)	0.994 (0.004)	0 (0)	0 (0)
26	0.914 (0.089)	0.986 (0.031)	0.256 (0.026)	0.994 (0.027)	0 (0)	0 (0)
27	0.889 (0.105)	0.872 (0.099)	0.667 (0.046)	0.998 (0.104)	0 (0)	0 (0)
28	0.939 (0.085)	0.954 (0.068)	0.636 (0.053)	0.996 (0.018)	0 (0)	0 (0)
29	0.344 (0.285)	0.189 (0.183)	0.145 (0.094)	0.991 (0.044)	0 (0)	0 (0)
30	0.200 (0)	0.315 (0.147)	0.2 (0)	0.2 (0)	0 (0)	0 (0)
31	0.199 (0)	0.266 (0.132)	0.199 (0)	0.232 (0.057)	0 (0)	0 (0)
32	0.908 (0.079)	0.725 (0.138)	0.628 (0.05)	0.973 (0.030)	0 (0)	0 (0)
33	0.850 (0.084)	0.63 (0.143)	0.55 (0.046)	0.881 (0.045)	0 (0)	0 (0)
34	0.880 (0.088)	0.688 (0.133)	0.602 (0.04)	0.895 (0.047)	0 (0)	0 (0)
35	0.989 (0)	0.999 (0.008)	0.837 (0.059)	1 (0)	0.038 (0.187)	0 (0)
36	0.937 (0.133)	1 (0)	0.558 (0.042)	1 (0)	0 (0)	0 (0)
37	0.982 (0.055)	1 (0)	0.971 (0.049)	0.999 (0.009)	0.557 (0.444)	0.05 (0.219)
38	0.976 (0.090)	0.998 (0.011)	0.85 (0.077)	0.997 (0.017)	0.053 (0.213)	0 (0)
39	0.733 (0.122)	0.652 (0.138)	0.399 (0.03)	0.829 (0.041)	0 (0)	0 (0)

Supplemental Table 6. Mean Hubert-Arabie Adjusted Rand Index (ARI_{HA}) (Standard Deviation) for LFR sparse count matrices. Note: LP is Label Propagation, NSA is Newman's Spectral Approach

Condition	NSA	Walktrap	Fast Modularity	Louvain GJA	LP	Infomap
1	0.358 (0.176)	0.662 (0.121)	0.664 (0.118)	0.667 (0.117)	0.646 (0.121)	0.668 (0.121)
2	0.866 (0.141)	0.945 (0.089)	0.914 (0.099)	0.936 (0.1)	0.874 (0.133)	0.928 (0.131)
3	0.365 (0.169)	0.657 (0.126)	0.658 (0.124)	0.656 (0.127)	0.648 (0.121)	0.663 (0.129)
4	0.853 (0.154)	0.942 (0.103)	0.896 (0.125)	0.933 (0.107)	0.859 (0.155)	0.882 (0.199)
5	0.645 (0.112)	0.885 (0.058)	0.847 (0.067)	0.884 (0.056)	0.861 (0.084)	0.71 (0.334)
6	0.661 (0.121)	0.892 (0.058)	0.827 (0.071)	0.865 (0.066)	0.832 (0.089)	0.596 (0.369)
7	0.789 (0.104)	0.984 (0.024)	0.924 (0.056)	0.978 (0.028)	0.967 (0.055)	0.977 (0.12)
8	0.777 (0.098)	0.989 (0.025)	0.934 (0.058)	0.976 (0.036)	0.962 (0.059)	0.893 (0.283)
9	0.949 (0.048)	1 (0)	0.974 (0.038)	1 (0)	0.991 (0.038)	0.997 (0.019)
10	0.944 (0.068)	1 (0)	0.973 (0.038)	1 (0)	0.99 (0.046)	0.999 (0.009)
11	0.436 (0.128)	0.999 (0.002)	0.818 (0.048)	0.915 (0.03)	0.996 (0.01)	0.88 (0.325)
12	0.472 (0.119)	1 (0.002)	0.76 (0.045)	0.831 (0.04)	0.993 (0.017)	0.593 (0.488)
13	0.642 (0.133)	1 (0)	0.851 (0.053)	0.954 (0.019)	0.997 (0.01)	1 (0)
14	0.691 (0.096)	1 (0)	0.827 (0.054)	0.918 (0.028)	0.997 (0.01)	0.999 (0.003)
15	0.812 (0.071)	1 (0)	0.859 (0.044)	0.994 (0.009)	0.999 (0.007)	1 (0)
16	0.847 (0.068)	1 (0)	0.867 (0.055)	0.983 (0.015)	0.999 (0.007)	1 (0)
17	0.493 (0.148)	1 (0)	0.807 (0.042)	0.964 (0.015)	0.998 (0.007)	1 (0)
18	0.575 (0.132)	1 (0)	0.774 (0.042)	0.902 (0.024)	0.997 (0.008)	1 (0.001)
19	0.473 (0.133)	1 (0)	0.409 (0.034)	0.902 (0.029)	0.985 (0.023)	1 (0)
20	0.474 (0.125)	1 (0)	0.397 (0.033)	0.793 (0.041)	0.983 (0.021)	0.998 (0.003)
21	0.681 (0.115)	1 (0)	0.802 (0.046)	0.987 (0.009)	0.999 (0.005)	1 (0)
22	0.746 (0.104)	1 (0)	0.797 (0.045)	0.959 (0.015)	0.998 (0.007)	1 (0)
23	0.522 (0.164)	1 (0)	0.419 (0.033)	0.95 (0.022)	0.988 (0.017)	1 (0)
24	0.473 (0.191)	1 (0)	0.395 (0.032)	0.889 (0.035)	0.99 (0.017)	1 (0)
25	0.942 (0.104)	0.941 (0.105)	0.948 (0.092)	0.94 (0.11)	0.949 (0.085)	0.928 (0.132)
26	0.777 (0.039)	0.998 (0.008)	1 (0.002)	1 (0)	0.839 (0.084)	1 (0)
27	0.436 (0.033)	0.992 (0.012)	0.991 (0.018)	1 (0)	0.636 (0.189)	1 (0)
28	0.416 (0.029)	0.98 (0.016)	0.991 (0.016)	1 (0)	0.569 (0.212)	1 (0)

Supplemental Table 7. Mean Modularity Ratio (Standard Deviation) for Correlations. Note: LP is Label Propagation, NSA is Newman's Spectral Approach

Condition	NSA	Walktrap	Fast Modularity	Louvain GJA	LP	Infomap
1	0.614 (0.077)	1 (0)	1 (0)	0.607 (0.077)	1 (0)	0.82 (0.386)
2	0.906 (0.080)	1 (0)	1 (0)	0.906 (0.079)	1 (0)	0.88 (0.327)
3	0.901 (0.041)	1 (0)	1 (0)	0.905 (0.073)	0.78 (0.416)	0.53 (0.502)
4	0.391 (0.066)	1.001 (0.011)	1.003 (0.006)	0.377 (0.067)	0.934 (0.163)	0.92 (0.241)
5	0.461 (0.071)	1 (0.012)	1.003 (0.008)	0.465 (0.070)	0.965 (0.116)	0.928 (0.256)
6	0.420 (0.061)	1.01 (0.014)	1.014 (0.01)	0.424 (0.061)	0.953 (0.138)	0.929 (0.252)
7	0.629 (0.054)	1 (0)	1 (0)	0.837 (0.024)	1 (0)	0.95 (0.219)
8	0.453 (0.047)	1 (0)	1 (0.001)	0.463 (0.046)	0.999 (0.011)	1 (0)
9	0.360 (0.023)	0.999 (0.004)	1 (0.001)	0.360 (0.023)	0.983 (0.053)	1 (0)
10	0.481 (0.046)	1.001 (0.005)	1.003 (0.004)	0.481 (0.045)	0.972 (0.079)	0.983 (0.114)
11	0.321 (0.029)	1 (0)	1 (0.002)	0.323 (0.028)	0.959 (0.12)	1 (0)
12	0.289 (0.026)	0.998 (0.012)	1.008 (0.007)	0.296 (0.045)	0.949 (0.143)	0.928 (0.258)
13	0.528 (0.034)	1 (0)	1 (0)	0.528 (0.034)	0.99 (0.1)	0.92 (0.273)
14	0.323 (0.023)	1 (0)	0.99 (0.007)	0.326 (0.022)	0.995 (0.022)	1 (0)
15	0.252 (0.026)	0.999 (0.003)	0.938 (0.016)	0.261 (0.024)	0.983 (0.101)	0.989 (0.101)
16	0.386 (0.021)	0.999 (0.004)	0.932 (0.018)	0.386 (0.021)	0.956 (0.172)	0.99 (0.1)
17	0.254 (0.020)	0.999 (0.005)	0.947 (0.017)	0.265 (0.020)	0.915 (0.232)	1.002 (0.003)
18	0.186 (0.015)	1 (0.001)	0.887 (0.024)	0.188 (0.014)	0.87 (0.309)	0.91 (0.288)
19	0.203 (0.017)	0.999 (0.011)	0.948 (0.021)	0.210 (0.017)	0.71 (0.456)	0.01 (0.101)
20	NaN	NaN	Nan	NaN	1 (0)	1 (0)
21	0.574 (0.030)	1 (0)	1 (0)	0.574 (0.0245)	0.99 (0.1)	1 (0)
22	0.418 (0.025)	1 (0)	1 (0.001)	0.427(0.025)	0.997 (0.022)	1 (0)
23	31.530 (428.498)	240.986 (3192.015)	316.07 (4258.719)	29.327 (389.00)	0 (0)	0 (0)
24	4.005 (0.007)	0.994 (0.051)	1.323 (0.048)	4.01 (0.138)	0 (0)	0 (0)
25	0.247 (0.001)	0.997 (0.004)	0.924 (0.013)	0.253 (0.016)	0.988 (0.1)	1 (0)
26	0.099 (0.006)	1 (0)	0.769 (0.027)	0.104 (0.006)	0 (0)	0 (0)
27	0.291 (0.022)	1 (0.001)	0.939 (0.015)	0306 (0.020)	0.997 (0.013)	1 (0)
28	0.455 (0.033)	1 (0.001)	0.918 (0.014)	0.465 (0.32)	0.146 (0.349)	0 (0)
29	0.722 (0.062)	0.995 (0.003)	0.799 (0.024)	0.853 (0.55)	0 (0)	0 (0)
30	0.325 (0.012)	1 (0)	0.935 (0.011)	0.325 (0.012)	0.997 (0.009)	1 (0)
31	0.358 (0.014)	1 (0)	0.934 (0.012)	0.354 (0.015)	0.998 (0.008)	1 (0)
32	0.305 (0.023)	1 (0.001)	0.948 (0.012)	0.317 (0.022)	0.993 (0.016)	0.99 (0.1)
33	0.354 (0.025)	1.005 (0.003)	0.984 (0.012)	0.362(0.0234)	0.968 (0.028)	1.001 (0.002)
34	0.375 (0.024)	1.004 (0.002)	0.982 (0.012)	03.78 (0.023)	0.949 (0.077)	1.001 (0.002)
35	0.430 (0.031)	1 (0)	0.967 (0.01)	0.437 (0.031)	0.998 (0.007)	1 (0)
36	0.218 (0.0169)	1 (0)	0.923 (0.014)	0.222 (0.014)	0.976 (0.103)	1 (0)
37	0.632 (0.045)	1 (0)	0.992 (0.006)	0.636 (0.045)	0.998 (0.008)	1 (0)
38	0.473 (0.035)	1 (0.001)	0.978 (0.01)	0.477 (0.0329)	0.994 (0.018)	1 (0)
39	0.249 (0.019)	1.002 (0.003)	0.919 (0.013)	0.257 (0.018)	0.978 (0.14)	0.871 (0.338)

Supplemental Table 8. Mean Modularity Ratio (Standard Deviation) for Reflected Euclidean Distance (EucD) matrices. Note: LP is Label Propagation, NSA is Newman's Spectral Approach

Condition	NSA	Walktrap	Fast Modularity	Louvain GJA	LP	Infomap
1	1.005 (0.006)	1 (0)	1 (0)	1.006 (0.006)	0.72 (0.451)	0 (0)
2	1 (0)	1 (0)	1 (0)	1 (0)	0.98 (0.141)	0.4 (0.492)
3	1.245 (0.037)	1 (0)	1 (0)	1.248 (0.037)	0.01 (0.1)	0 (0)
4	1.014 (0.01)	0.992 (0.04)	1.01 (0.015)	1.019 (0.008)	0.062 (0.23)	0 (0)
5	0.999 (0.004)	0.969 (0.047)	1.008 (0.014)	1.001 (0.002)	0.13 (0.314)	0 (0)
6	0.998 (0.005)	0.992 (0.043)	1.034 (0.021)	1 (0.001)	0.165 (0.355)	0 (0)
7	1 (0)	1 (0)	1 (0)	1 (0)	0.65 (0.479)	0 (0)
8	0.985 (0.022)	0.995 (0.013)	1 (0.001)	1 (0)	0.169 (0.375)	0 (0)
9	1.233 (0.016)	1 (0)	1.432 (0.018)	1.233 (0.016)	0 (0)	0 (0)
10	1 (0.003)	0.996 (0.015)	1.004 (0.005)	1.001 (0.002)	0.107 (0.292)	0 (0)
11	0.997 (0.008)	0.998 (0.011)	0.997 (0.012)	1 (0)	0.009 (0.094)	0 (0)
12	0.988 (0.011)	0.913 (0.064)	1.018 (0.011)	1 (0.001)	0 (0)	0 (0)
13	1 (0)	1 (0)	1 (0)	1 (0)	0.27 (0.446)	0 (0)
14	0.993 (0.016)	0.995 (0.015)	0.98 (0.014)	1 (0)	0 (0)	0 (0)
15	0.971 (0.035)	0.944 (0.046)	0.93 (0.016)	1 (0.001)	0 (0)	0 (0)
16	2.793 (0.012)	2.721 (0.282)	3.169 (0.012)	2.793 (0.012)	0 (0)	0 (0)
17	0.966 (0.036)	0.87 (0.06)	0.939 (0.016)	1.004 (0.003)	0 (0)	0 (0)
18	0.993 (0.025)	1.001 (0.003)	0.85 (0.03)	1.001 (0.001)	0 (0)	0 (0)
19	0.986 (0.016)	0.905 (0.038)	0.947 (0.017)	1.018 (0.005)	0 (0)	0 (0)
20	NaN	NAN	NAN	NaN	1 (0)	1 (0)
21	1 (0)	1 (0)	1 (0)	1 (0)	0.53 (0.502)	0 (0)
22	1 (0)	1 (0)	0.999 (0.001)	1 (0)	0.09 (0.288)	0 (0)
23	0.874 (0.069)	-0.005 (0.007)	-4.614 (0.519)	1 (0.065)	0 (0)	0 (0)
24	2.928 (0.004)	2.886 (0.038)	3.34 (0.005)	2.928 (0.004)	0 (0)	0 (0)
25	0.99 (0.001)	0.878 (0.033)	0.904 (0.017)	1 (0)	0 (0)	0 (0)
26	0.881 (0.009)	1 (0.001)	0.704 (0.035)	0.997 (0.008)	0 (0)	0 (0)
27	0.956 (0.001)	0.966 (0.033)	0.933 (0.013)	1 (0)	0 (0)	0 (0)
28	0.999 (0)	0.995 (0.01)	0.927 (0.015)	1 (0)	0 (0)	0 (0)
29	0.963 (0.006)	0.659 (0.109)	0.714 (0.05)	0.999 (0)	0 (0)	0 (0)
30	2.76 (0.01)	2.633 (0.324)	3.13 (0.009)	2.76 (0.01)	0 (0)	0 (0)
31	2.504 (0.002)	2.426 (0.186)	2.782 (0.002)	2.496 (0.011)	0 (0)	0 (0)
32	0.946 (0.001)	0.947 (0.038)	0.936 (0.011)	0.996 (0.003)	0 (0)	0 (0)
33	1.009 (0.004)	0.97 (0.04)	0.96 (0.014)	1.01 (0.004)	0 (0)	0 (0)
34	0.997 (0.002)	0.977 (0.034)	0.969 (0.014)	1.004 (0.003)	0 (0)	0 (0)
35	0.987 (0)	1 (0.001)	0.977 (0.009)	1 (0)	0.04 (0.195)	0 (0)
36	0.78 (0.012)	1 (0)	0.899 (0.017)	1 (0)	0 (0)	0 (0)
37	1 (0)	1 (0)	0.998 (0.004)	1 (0)	0.608 (0.478)	0.05 (0.219)
38	0.929 (0.009)	1 (0)	0.984 (0.009)	1 (0)	0.059 (0.234)	0 (0)
39	0.949 (0.006)	0.944 (0.029)	0.921 (0.012)	0.983 (0.006)	0 (0)	0 (0)

Supplemental Table 9. Mean Modularity Ratio (Standard Deviation) for LFR sparse count matrices. Note: LP is Label Propagation, NSA is Newman's Spectral Approach

Condition	NSA	Walktrap	Fast Modularity	Louvain GJA	LP	Infomap
1	0.669 (0.273)	1.149 (0.103)	1.149 (0.103)	1.15 (0.102)	1.127 (0.109)	1.146 (0.105)
2	0.961 (0.061)	1.005 (0.019)	0.996 (0.023)	1.003 (0.02)	0.978 (0.041)	0.994 (0.047)
3	0.699 (0.293)	1.16 (0.115)	1.16 (0.114)	1.16 (0.114)	1.142 (0.12)	1.158 (0.114)
4	0.941 (0.097)	1.003 (0.013)	0.989 (0.029)	0.997 (0.018)	0.963 (0.069)	0.974 (0.083)
5	0.754 (0.092)	1.003 (0.008)	1.003 (0.01)	1.008 (0.007)	0.986 (0.028)	0.85 (0.261)
6	0.769 (0.107)	1.003 (0.006)	1.007 (0.014)	1.013 (0.008)	0.984 (0.022)	0.784 (0.266)
7	0.823 (0.096)	1 (0.003)	0.991 (0.011)	1.001 (0.002)	0.992 (0.021)	0.987 (0.091)
8	0.83 (0.092)	1 (0.003)	0.993 (0.012)	1.001 (0.003)	0.992 (0.016)	0.925 (0.213)
9	0.949 (0.047)	1 (0)	0.987 (0.018)	1 (0)	0.997 (0.016)	0.999 (0.005)
10	0.943 (0.062)	1 (0)	0.988 (0.017)	1 (0)	0.996 (0.022)	1 (0.001)
11	0.569 (0.063)	1 (0)	0.987 (0.008)	1.003 (0.001)	1 (0.002)	0.885 (0.312)
12	0.62 (0.062)	1 (0)	0.993 (0.008)	1.005 (0.001)	1 (0.002)	0.637 (0.44)
13	0.706 (0.086)	1 (0)	0.985 (0.008)	1.001 (0)	1 (0.001)	1 (0)
14	0.755 (0.072)	1 (0)	0.99 (0.007)	1.001 (0.002)	1 (0.001)	1 (0)
15	0.836 (0.048)	1 (0)	0.983 (0.007)	1 (0)	1 (0)	1 (0)
16	0.877 (0.045)	1 (0)	0.986 (0.007)	1 (0)	1 (0.001)	1 (0)
17	0.604 (0.067)	1 (0)	0.981 (0.005)	1.001 (0)	1 (0.001)	1 (0)
18	0.686 (0.064)	1 (0)	0.987 (0.005)	1.001 (0)	1 (0.001)	1 (0)
19	0.759 (0.047)	1 (0)	0.908 (0.011)	1.002 (0.001)	0.999 (0.002)	1 (0)
20	0.805 (0.051)	1 (0)	0.928 (0.011)	1.005 (0.002)	1 (0.001)	1 (0)
21	0.726 (0.065)	1 (0)	0.98 (0.005)	1 (0)	1 (0)	1 (0)
22	0.796 (0.065)	1 (0)	0.985 (0.005)	1 (0)	1 (0)	1 (0)
23	0.818 (0.044)	1 (0)	0.907 (0.011)	1.001 (0.001)	0.999 (0.001)	1 (0)
24	0.833 (0.046)	1 (0)	0.916 (0.011)	1.001 (0.001)	1 (0.001)	1 (0)
25	0.974 (0.058)	0.973 (0.059)	0.975 (0.055)	0.973 (0.059)	0.976 (0.055)	0.972 (0.065)
26	0.98 (0.005)	1 (0.001)	1 (0)	1 (0)	0.884 (0.036)	1 (0)
27	0.903 (0.01)	1 (0)	0.999 (0.001)	1 (0)	0.877 (0.051)	1 (0)
28	0.908 (0.01)	1 (0.001)	1 (0.001)	1 (0)	0.873 (0.05)	1 (0)

Supplemental Table 10. Effect Sizes (d) for aggregate comparisons between algorithms in terms of the Hubert-Arabie Adjusted Rand Index (ARI_{HA}) for LFR count matrices. Negative values indicate the column header performed better than the algorithm listed in the row in terms of recovery of the simulated true communities.
Note: “NSA” is Newman’s Spectral Approach

**LFR SparseCount**	*NSA*	*Walktrap*	*Fast Modularity*	*Louvain GJA*	*LP*
Walktrap	1.82	-	-	-	-
Fast Modularity	.42	-1.40	-	-	-
Louvain GJA	1.57	-.40	1.13	-	
LP	1.69	-.14	1.27	.24	-
Infomap	1.23	-.25	.86	.00	-.16

Supplemental Table 11. Effect Sizes (d) for aggregate comparisons between algorithms in terms of the Hubert-Arabie Adjusted Rand Index (ARI_{HA}) for correlation and Euclidean distances. Note: “NSA” is Newman’s Spectral Approach; “cor” and “euc” within title reflects correlation and reflected Euclidean Distance matrices, respectively. Values displayed reflect the average for the rows subtracted by columns.

Method	NSAcorr	NSAeuc	Walkcorr	Walkeuc	Fastcorr	Fasteuc	GJAcorr	GJAeuc	Labelcorr	Labeleuc	Infocorr	Infoeuc
NSAcorr	0.00	-0.27	0.20	-0.40	-0.50	-0.72	0.06	-0.19	-0.18	-2.36	-0.19	-3.52
NSAeuc	0.27	0.00	0.46	-0.12	-0.20	-0.42	0.33	0.07	0.07	-1.96	0.05	-2.83
Walkcorr	-0.20	-0.46	0.00	-0.59	-0.71	-0.93	-0.13	-0.37	-0.35	-2.63	-0.35	-4.00
Walkeuc	0.40	0.12	0.59	0.00	-0.08	-0.30	0.45	0.19	0.19	-1.84	0.15	-2.69
Fastcorr	0.50	0.20	0.71	0.08	0.00	-0.24	0.56	0.27	0.27	-1.84	0.23	-2.77
Fasteuc	0.72	0.42	0.93	0.30	0.24	0.00	0.77	0.48	0.47	-1.54	0.42	-2.29
GJAcorr	-0.06	-0.33	0.13	-0.45	-0.56	-0.77	0.00	-0.25	-0.23	-2.40	-0.24	-3.54
GJAeuc	0.19	-0.07	0.37	-0.19	-0.27	-0.48	0.25	0.00	0.00	-2.00	-0.02	-2.87
Labelcorr	0.18	-0.07	0.35	-0.19	-0.27	-0.47	0.23	0.00	0.00	-1.94	-0.02	-2.73
Labeleuc	2.36	1.96	2.63	1.84	1.84	1.54	2.40	2.00	1.94	0.00	1.81	-0.37
Infocorr	0.19	-0.05	0.35	-0.15	-0.23	-0.42	0.24	0.02	0.02	-1.81	0.00	-2.48
Infoeuc	3.52	2.83	4.00	2.69	2.77	2.29	3.54	2.87	2.73	0.37	2.48	0.00

Supplemental Table 12. Average deviation in solution recovery across data sets per condition (Standard Deviation) for Correlation (Corr) and Reflected Euclidean Distance (EucD) matrices conducted on Louvain GJA and Label Propagation ("Label Prop").

Condition	Louvain GJA Corr	Louvain GJA EucD	Label Prop Corr	Label Prop EucD
1	0 (0)	0 (0)	0.058 (0.082)	0.421 (0.071)
2	0 (0)	0 (0)	0.048 (0.07)	0.158 (0.087)
3	0.04 (0.023)	0.005 (0.004)	0.31 (0.145)	0.005 (0.046)
4	0 (0)	0.003 (0.011)	0.121 (0.065)	0.123 (0.096)
5	0 (0)	0 (0)	0.078 (0.051)	0.151 (0.11)
6	0 (0)	0 (0)	0.129 (0.081)	0.099 (0.077)
7	0 (0)	0 (0)	0.052 (0.071)	0.446 (0.046)
8	0 (0)	0 (0)	0.066 (0.035)	0.35 (0.077)
9	0 (0)	0 (0)	0.081 (0.032)	0 (0)
10	0 (0)	0 (0)	0.08 (0.047)	0.16 (0.064)
11	0 (0)	0 (0)	0.136 (0.041)	0.028 (0.041)
12	0 (0.001)	0 (0)	0.175 (0.078)	0 (0)
13	0 (0)	0 (0)	0.082 (0.082)	0.479 (0.03)
14	0 (0)	0 (0)	0.075 (0.032)	0.009 (0.032)
15	0 (0)	0 (0)	0.148 (0.068)	0 (0)
16	0 (0.002)	0 (0)	0.157 (0.061)	0.002 (0.005)
17	0.002 (0.006)	0.001 (0.004)	0.189 (0.062)	0 (0)
18	0.003 (0.005)	0.001 (0.003)	0.306 (0.063)	0 (0)
19	0.014 (0.011)	0.011 (0.011)	0.404 (0.051)	0 (0)
20	0 (0)	0 (0)	0 (0)	0 (0)
21	0 (0)	0 (0)	0.035 (0.053)	0.488 (0.018)
22	0 (0)	0 (0)	0.042 (0.024)	0.249 (0.066)
23	0.001 (0)	0.001 (0)	0 (0)	0 (0)
24	0.01 (0.002)	0 (0)	0 (0)	0.001 (0.004)
25	0 (0)	0 (0)	0.09 (0.039)	0 (0)
26	0.033 (0.006)	0.031 (0.005)	0.007 (0.024)	0 (0)
27	0 (0)	0 (0)	0.063 (0.023)	0 (0)
28	0 (0)	0 (0)	0.318 (0.068)	0 (0)
29	0.001 (0.003)	0.006 (0.027)	0 (0)	0 (0)
30	0 (0)	0 (0)	0.051 (0.018)	0 (0.003)
31	0 (0)	0.059 (0.003)	0.053 (0.022)	0.003 (0.007)
32	0 (0)	0 (0)	0.101 (0.046)	0 (0)
33	0.004 (0.008)	0.007 (0.009)	0.1 (0.033)	0 (0)
34	0.004 (0.006)	0.007 (0.011)	0.107 (0.033)	0 (0)
35	0 (0)	0 (0)	0.053 (0.02)	0.194 (0.077)
36	0 (0.001)	0 (0.001)	0.158 (0.046)	0 (0)
37	0 (0)	0 (0)	0.064 (0.028)	0.438 (0.029)
38	0 (0)	0 (0)	0.086 (0.028)	0.205 (0.066)
39	0.005 (0.008)	0.003 (0.007)	0.191 (0.063)	0 (0)

Supplemental Table 13. Average deviation in solution recovery across data sets per condition (Standard Deviation) for LFR matrices conducted on Louvain GJA and Label Propagation ("Label Prop").

Condition	Louvain GJA	Label Prop
1	0.005 (0.017)	0.05 (0.029)
2	0.019 (0.031)	0.11 (0.03)
3	0.006 (0.017)	0.046 (0.031)
4	0.024 (0.04)	0.116 (0.029)
5	0.01 (0.011)	0.063 (0.012)
6	0.008 (0.012)	0.067 (0.015)
7	0.002 (0.004)	0.047 (0.013)
8	0.001 (0.003)	0.052 (0.013)
9	0 (0)	0.03 (0.012)
10	0 (0)	0.029 (0.011)
11	0.004 (0.004)	0.012 (0.003)
12	0.009 (0.004)	0.014 (0.003)
13	0.002 (0.003)	0.009 (0.003)
14	0.004 (0.004)	0.01 (0.003)
15	0 (0.001)	0.007 (0.003)
16	0.001 (0.003)	0.008 (0.003)
17	0.002 (0.002)	0.005 (0.002)
18	0.006 (0.003)	0.007 (0.002)
19	0.01 (0.003)	0.021 (0.004)
20	0.014 (0.003)	0.025 (0.004)
21	0.001 (0.002)	0.005 (0.002)
22	0.003 (0.003)	0.006 (0.002)
23	0.008 (0.003)	0.019 (0.004)
24	0.013 (0.004)	0.021 (0.004)
25	0 (0.002)	0.004 (0.002)
26	0.003 (0.004)	0.004 (0.002)
27	0.007 (0.003)	0.017 (0.003)
28	0.011 (0.004)	0.018 (0.003)

Figures

Figure 1: **Majority Placement for LFR Sparse Matrices.**

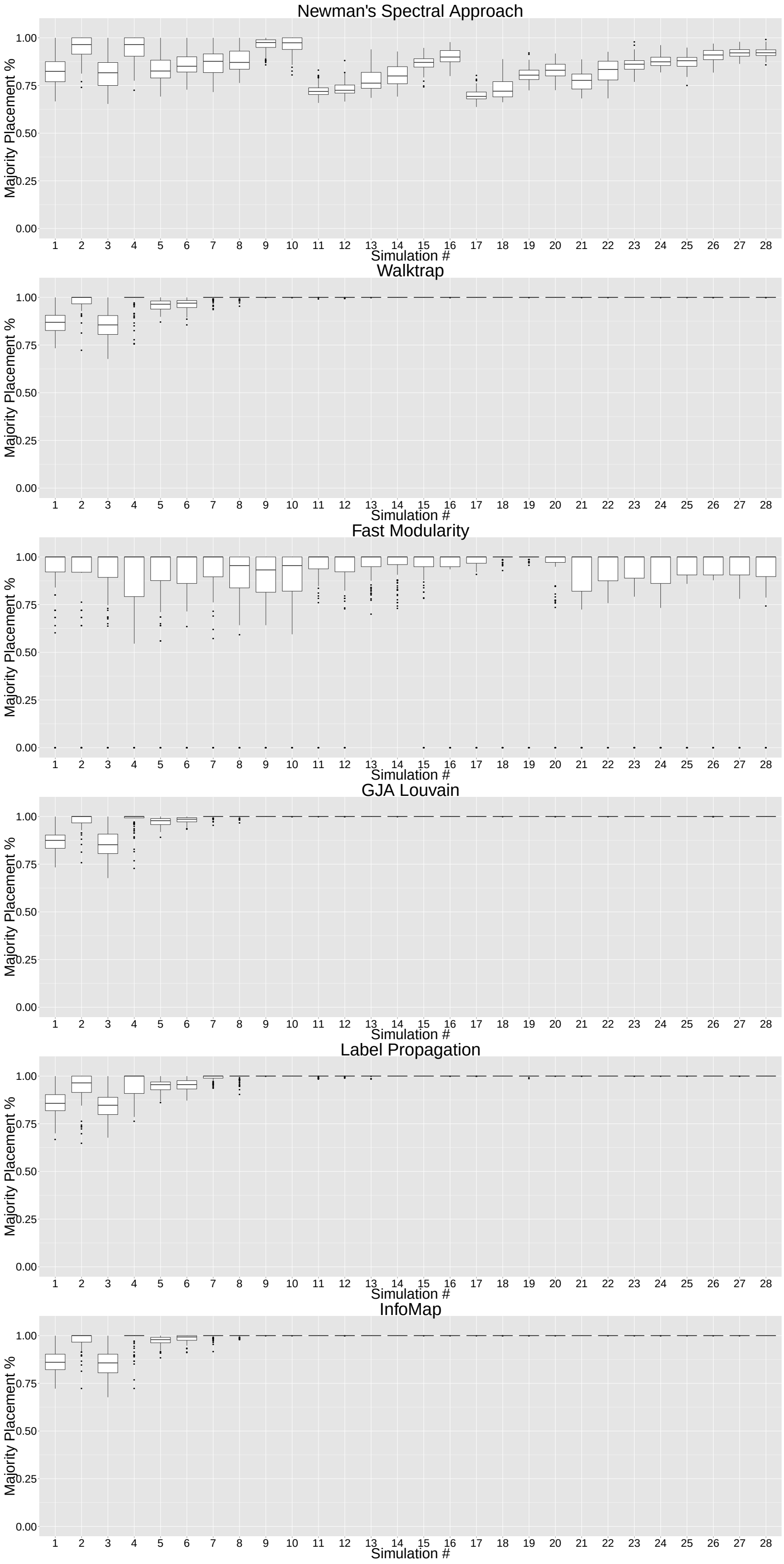

Figure 2: **Modularity Ratio for LFR Sparse Matrices.**

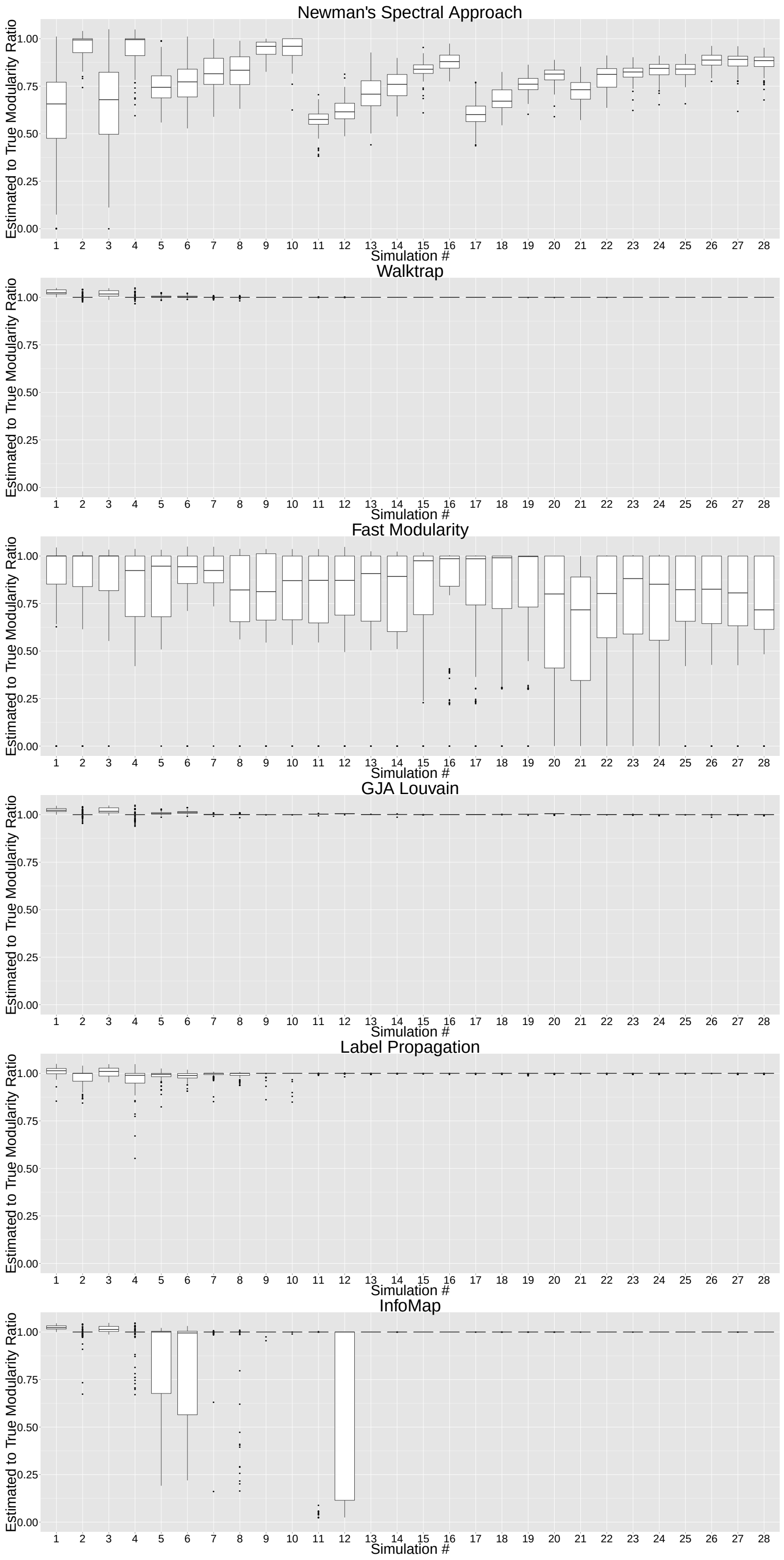

Supplement: Supplementary file 1 [file DataSheet1.pdf]
